# Age-related changes in the architecture and biochemical markers levels in motor-related cortical areas of SHR rats—an ADHD animal model

**DOI:** 10.3389/fnmol.2024.1414457

**Published:** 2024-08-23

**Authors:** E. Bogdańska-Chomczyk, P. Wojtacha, M. L Tsai, A. C. W Huang, A. Kozłowska

**Affiliations:** ^1^Department of Human Physiology and Pathophysiology, School of Medicine, Collegium Medicum, University of Warmia and Mazury in Olsztyn, Olsztyn, Poland; ^2^Department of Psychology and Sociology of Health and Public Health, University of Warmia and Mazury in Olsztyn, Olsztyn, Poland; ^3^Department of Biomechatronic Engineering, National Ilan University, Yilan, Taiwan; ^4^Department of Psychology, Fo Guang University, Yilan, Taiwan

**Keywords:** rat, ADHD, motor cortex abnormalities, SHR, brain maturation, neuron density

## Abstract

**Introduction:**

Attention-deficit/hyperactivity disorder (ADHD) is a neurodevelopmental disorder whose exact pathophysiology has not been fully understood yet. Numerous studies have suggested disruptions in the cellular architecture and neuronal activity within brain structures of individuals with ADHD, accompanied by imbalances in the immune system, oxidative stress, and metabolism.

**Methods:**

This study aims to assess two functionally and histologically distinct brain areas involved in motor control and coordination: the motor cortex (MC) and prefrontal cortex (PFC). Namely, the morphometric analysis of the MC throughout the developmental stages of Spontaneously Hypertensive Rats (SHRs) and Wistar Kyoto Rats (WKYs). Additionally, the study aimed to investigate the levels and activities of specific immune, oxidative stress, and metabolic markers in the PFC of juvenile and maturing SHRs in comparison to WKYs.

**Results:**

The most significant MC volume reductions occurred in juvenile SHRs, accompanied by alterations in neuronal density in these brain areas compared to WKYs. Furthermore, juvenile SHRs exhibit heightened levels and activity of various markers, including interleukin-1α (IL-1α), IL-6, serine/threonine-protein mammalian target of rapamycin, RAC-alpha serine/threonine-protein kinase, glucocorticoid receptor β, malondialdehyde, sulfhydryl groups, superoxide dismutase, peroxidase, glutathione reductase, glutathione S-transferase, glucose, fructosamine, iron, lactic acid, alanine, aspartate transaminase, and lactate dehydrogenase.

**Discussion:**

Significant changes in the MC morphometry and elevated levels of inflammatory, oxidative, and metabolic markers in PFC might be associated with disrupted brain development and maturation in ADHD.

## 1 Introduction

Attention-deficit/hyperactivity disorder (ADHD) represents a prevalent and chronic neurodevelopmental condition characterized by symptoms of hyperactivity, impulsivity, and age-inappropriate inattention. These manifestations significantly impact various domains of life, including social interactions, academic performance, and occupational functioning (Sibley et al., 2016). The global prevalence of ADHD is estimated to be approximately 7.6% in children and 5.6% in adolescents (Salari et al., 2023). Moreover, the persistence of this disorder symptoms into adulthood is observed in approximately 75% of individuals (Sibley et al., 2012). It is noteworthy that ADHD diagnoses exhibit a notable gender disparity, with males being diagnosed three times more frequently than females (Gaub and Carlson, 1997; Ramtekkar et al., 2010). Despite the abundant research findings, ADHD often goes undiagnosed and untreated, frequently coexisting with other mental health disorders such as major depression, personality disorders, and addictions, thereby posing severe health and social problems (Elia et al., 2008; Naya et al., 2021). In the course of ADHD, disrupted dopaminergic, noradrenergic, glutamatergic, and serotonergic neurotransmission have been observed (Pliszka, 2005). These deficiencies are associated with structural anomalies in cortical and subcortical brain areas, observed both in individuals with ADHD and in spontaneously hypertensive rats (SHRs), the most well-validated model of this disorder (Sagvolden et al., 2009). Current studies indicate delayed brain maturation in ADHD patients and total brain volume reduction in white matter particularly in the areas of prefrontal cortex (PFC), corpus callosum, cingulate gyrus and internal capsule. Moreover, gray matter reduction was also observed in PFC, striatum, cerebellum, anterior cingulate cortex, hippocampus, and amygdala. It is worth mentioning that the most remarkable differences have been observed in the right brain hemisphere (Almeida et al., 2010; Li et al., 2019; Ha et al., 2020). Due to the variety of neuroanatomical alterations observed in several brain areas, ADHD is a complex condition. This complexity is evident in the wide range of symptoms of inattention, hyperactivity and impulsivity and also social and mood-related behaviors. One of the most prominent symptoms of ADHD is excessive motor activity. Therefore, this manuscript will focus on alternations in the motor cortex areas (M1 and M2) that may lead to the deficits in motor control and coordination associated with ADHD.

The motor cortex (MC) plays a key role in the regulation of motor inhibition and impulsive actions. Current literature suggests neuroanatomical and functional alterations in the MC areas in children with ADHD, which contribute to the hyperactivity and motor control difficulties often observed in people with this disorder (Gilbert et al., 2019). For example, several studies have shown reduced activation of the MC during motor tasks in children with ADHD compared to typically developing individuals, which may reflect inefficiencies in motor planning and execution processes (Mostofsky et al., 2006; Paloyelis et al., 2007). Moreover, magnetic resonance imaging studies (MRI) have reported alterations in the thickness of the MC in individuals with ADHD, although findings have been inconsistent across studies (Sutcubasi Kaya et al., 2018; Hoogman et al., 2019). It is worth adding that research suggests that individuals with ADHD may exhibit reduced cortical excitability within the MC, which may affect motor performance and control (Dutra et al., 2016).

In rodents, the MC is divided into two closely cooperating regions: the primary motor cortex (M1) and secondary motor cortex (M2), differing in cytoarchitectonic structure and functions (Soma et al., 2017). M1, located in the precentral gyrus of the brain’s frontal lobe, is renowned as the central hub for motor decisions and movements, with its axons following pyramidal pathways to spinal cord movement centers, connecting with neurons innervating muscles (Lee et al., 2022). M2, adjacent to M1, strongly associates with the cerebellum and basal ganglia (Bostan et al., 2013). Similar to M1, M2 features a somatotopic organization of neurons (Barthas and Kwan, 2017) and plays a primary role in planning voluntary movements, collaborating with other brain regions for motor activity coordination (Liu et al., 2023). Importantly, the neurons in both M1 and M2 exhibit a structured organization across six parallel layers. Each layer is characterized by different number and types of nerve cells and the connections that are received and sent to other brain structures (Imai et al., 2012), one of these structures is PFC. At first, it should be emphasized that the PFC is a functionally and histologically distinct structure. Nevertheless, the relationship between the PFC and the MC in motor control and ADHD symptoms is complex and multifaceted. PFC is involved in executive functions such as decision-making, impulse control, attention regulation and working memory. It plays a key role in the regulation of behavior and cognitive processes. It sends signals to the MC to initiate, inhibit or modify voluntary movements based on cognitive processes such as attention, planning and decision-making (Grafton and Volz, 2019). More precisely, neural tract tracing studies in the rats showed multiple projections from M1 and M2 to the PFC and from the PFC to M2 (Muñoz-Castañeda et al., 2021; Bedwell et al., 2023). Similar projections were also found in a human brain using functional magnetic resonance imaging (fMRI) study (Schellekens et al., 2022). Moreover, the PFC damage in rodents and primates caused a range of motor impairments, including hyperactivity and increased aimless locomotion, further confirming the dependencies between these cortices (Fuster, 2002; Szczepanski and Knight, 2014). Additionally, it has been shown that PFC injury is associated with a range of symptoms seen in ADHD including difficulties in concentrating, impulsivity, and hyperactivity. Both conditions can result in problems with executive functioning, such as planning, organizing tasks, and regulating emotions (Szczepanski and Knight, 2014; Fremont et al., 2022).

Recent literature emphasizes a strong association between immune system activation and ADHD pathophysiology (Corona, 2020; Wong, 2022). It is generally known that cytokines are a broad group of immune system proteins involved in inflammatory reactions that affect brain function, structure, and development (Miller et al., 2013). For example, our previous studies on SHRs, a validated ADHD animal model, have revealed ongoing inflammatory processes affecting brain morphology and neurotransmitter levels (Kozłowska et al., 2019a,b). Correspondingly, increased serum levels of interleukin-6 (IL-6) and interleukin-10 (IL-10) have been reported in children with ADHD (Donfrancesco et al., 2020). Furthermore, prolonged inflammation and immune system disturbances can lead to heightened oxidative stress observed in ADHD patients (Leffa et al., 2017; Corona, 2020) and SHRs (Kozłowska et al., 2019b). Oxidative stress involves an imbalance between radical production and the antioxidative system. This state lead to neuronal damage by disrupting membrane integrity causing DNA and lipids damage, and protein oxidation (Guo et al., 2013). Oxidative stress and neuroinflammation can intricately impact brain metabolism (Zia et al., 2021). Neurometabolic studies on adults with ADHD show approximately 8.3% reduction in brain glucose metabolism (Zametkin et al., 1990) and alterations in biochemical markers like ferritin, 3-indole propionic acid, kynurenic acid, and lactate dehydrogenase (LDH) (Juneja et al., 2010; Puurunen et al., 2016; Zhou et al., 2017). Despite numerous studies on ADHD’s association with changes in brain architecture, neurotransmission, immune, oxidative stress, and metabolic markers, some data remain inconsistent, overlooking developmental changes and requiring further investigation. Certainly, to better understand the pathophysiology of ADHD, detailed findings that consider developmental differences are needed. Such an approach would enable significant advances in finding the pathophysiology of ADHD. Human studies are very difficult (sometimes impossible) and time consuming to obtain the above-discussed data. Therefore, using an animal model, particularly male SHRs, offers a valuable avenue for exploring ADHD pathophysiology. It is worth mentioning that the characterization of this animal model is based on numerous findings that have confirmed that SHR rats manifest key symptoms of ADHD, such as attention deficit, hyperactivity, impulsivity, and deficits in learning and memory, at specific age stages (Johansen et al., 2005, 2007; Kim et al., 2024). It has been shown that this strain is similar to children with ADHD because they are more sensitive to delayed reinforcement, which is in line with the steeper gradient of delayed reinforcement observed in SHRs in comparison to controls (Johansen et al., 2005, 2007). Furthermore, SHRs showed alterations in several brain systems, including the dopaminergic and noradrenergic systems. Previous findings have demonstrated reduced dopamine (DA) release in SHRs in key brain areas involved in the pathophysiology of ADHD: the PFC, caudate-striatal nucleus, and paraventricular nucleus (Leffa et al., 2018, 2019). Additionally, norepinephrine production in the PFC was higher in SHRs compared to controls (Leffa et al., 2019). This evidence suggests that SHR rats are the most established and best-validated model of ADHD.

Building upon these findings, our hypothesis suggests that individuals affected by ADHD demonstrate abnormalities in the areas of the brain associated with motor control and coordination: (a) a different trajectory of volumetric alterations in M1 and M2 during postnatal development compared to control animals; (b) differences in comparison to the typical developmental pattern of neuronal distribution in different layers in both M1 and M2 as well as; (c) disruptions in immune homeostasis, oxidative stress equilibrium, and distinct metabolic profiles within the PFC when compared to controls.

To examine these hypotheses, we conducted a comprehensive morphometric analysis, encompassing measurements of both volume and neuron density (across distinct layers) of the M1 and M2 implicated in motor control and coordination. This analysis spanned all developmental stages of SHRs and Wistar Kyoto Rats (WKYs) from 4 to 10 weeks. Additionally, we scrutinized the content/activity of specific immunological, oxidative stress, and metabolic markers in the PFC of juvenile (5-week-old) and maturing (10-week-old) SHRs. All experimental procedures were carried out using male SHRs, considered the widely accepted and best-validated animal model for ADHD, with WKYs serving as a control strain during postnatal development (Sagvolden and Johansen, 2012). The inclusion of male SHRs aligns with the significant male bias in the prevalence of ADHD (Gaub and Carlson, 1997). Additionally, the study incorporates an analysis of the hierarchical clustering and functional importance of mutual interactions of the studied protein markers using the STRING database (Szklarczyk et al., 2015).

## 2 Materials and methods

### 2.1 Experimental procedures

#### 2.1.1 Animals

The selection of animal strains was carefully considered to guarantee the utmost reliability in the outcomes. Furthermore, it is crucial to emphasize that the same rat strains underwent extensive examination in our previous investigations (Hsu et al., 2010; Tsai et al., 2017; Kozłowska et al., 2019a,b,c). Importantly, no statistically significant differences in the average body weight were observed between the experimental group - inbred Spontaneously Hypertensive Rats (SHR/NCrl) and the control group - inbred Wistar Kyoto Rats (WKY/NCrl) at equivalent developmental stages (Supplementary material 1). Significantly, an equal number of sections per animal were consistently examined, contingent upon the specific cortex region and developmental stage under investigation. The reference points for initiating and concluding the assessment of each brain remained consistent throughout the study.

##### 2.1.1.1 Animals with transcardial perfusion

To assess changes in brain morphometry during ADHD development, this experiment involved two cohorts of male rats: thirty-eight WKY/NCrl as a control group and thirty-eight SHR/NCrl as an animal model for ADHD. Analyses were conducted on animals (WKY/NCrl and SHR/NCrl) aged 4, 5, 6, 7, 8, 9, and 10 weeks seven distinct age stages; *n* = 5–6 (for each strain at each age). Three-week-old rats of both strains were purchased from Charles River Laboratory Germany GmbH (Sulzfeld, Germany) and transported to the animal house at the Institute of Animal Reproduction and Food Research of the Polish Academy of Sciences in Olsztyn (Olsztyn, Poland). Both SHR/NCrl and WKY/NCrl were acclimatized and housed in sanitized polypropylene cages in groups of three to mitigate isolation stress. The animal room maintained a consistent temperature (21 ± 1°C), ventilation (12–20 exchanges/h), and a light/dark cycle (12/12 h). All animals received the same VRF1 diet (Charles River Laboratories, Germany) and had unrestricted access to tap water. Ethical approval for all animal procedures was granted by the Local Ethics Committee for Animal Experimentation at the University of Warmia and Mazury in Olsztyn, Poland (permission number: no. 43/2014). Stringent adherence to the European Union Directive for animal experiments (2010/63/EU) ensured proper animal care and handling, emphasizing efforts to minimize suffering and the use of the minimum number of animals necessary for accurate scientific data.

##### 2.1.1.2 Animals without transcardial perfusion

Twenty-four rats, at the age of 3 weeks, were acquired from Charles River Laboratory Germany GmbH (Sulzfeld, Germany) and then relocated to the animal facility at the Institute of Animal Reproduction and Food Research of the Polish Academy of Sciences in Olsztyn (Olsztyn, Poland) to investigate differences in immune, oxidative stress and metabolic markers in the PFC between juvenile and maturing male WKY/NCrl (*n* = 12) and SHR/NCrl (*n* = 12). The standardized conditions for housing, nutrition, and hydration, as previously outlined, were consistently maintained for all animals. Analyses were conducted on 5-week-old (juvenile animals) and 10-week-old (maturing animals) rats in each group (WKY/NCrl and SHR/NCrl; 6 animals per group). The animal care and handling strictly adhered to the European Union Directive on animal experimentation (2010/63/EU) and the 3Rs principles- replacement, reduction, and refinement. All the animals used were registered, and personnel received appropriate training (certificate no. 1387/2015). The study’s reporting followed ARRIVE guidelines. All efforts were made to minimize animal suffering and distress.

#### 2.1.2 Preparation of brains for neuron-specific nuclear protein (NeuN) – staining

Following a one-week habituation phase, all SHR/NCrl and WKY/NCrl underwent the same experimental procedures. The rats were deeply anesthetized with an intraperitoneal injection of pentobarbital (Morbital, Biowet, Poland; 50 mg/kg body weight) according to the outlines of the Humane Society Veterinary Medical Association. After cessation of breathing, they were directly perfused transcardially with 0.9% saline, followed afterward, 4% paraformaldehyde (PFA; pH 7.4; 1,040,051,000, Merck, Germany) in phosphate-buffered saline (PBS; P5493, Sigma-Aldrich, Darmstadt, Germany) to preserve the natural state of the tissue. Subsequently, the brains were dissected from the skulls and post-fixed in 4% PFA for 24 h, washed three times in 0.1 M PBS (pH = 7.4, 4°C), and then cryoprotected for 4 days in graded solutions (10%, 20%, 30%) of sucrose (363–117,720,907, ALCHEM, Poland) at 4°C. Finally, coronal sections 10 μm thick were cut with a cryostat (HM525 Zeiss, Germany). The brain sections were mounted on object slides and stored at −80°C until further tissue processing.

#### 2.1.3 Preparation of brains for measuring the content of inflammatory, oxidative stress, and metabolic markers

After the habituation phase (7 days), the animals (*n* = 24) were divided into the following groups: 1) control 5-week-old WKY/NCrl (juvenile animals); 2) control 10-week-old WKY/NCrl (maturing animals); 3) ADHD 5-week-old SHR/NCrl (juvenile animals); 4) ADHD 10-week-old SHR/NCrl (maturing animals). All animals were anesthetized with 100 mg/kg body weight (BW) ketamine and 10 mg/kg BW xylazine according to the recommendations for the euthanasia of experimental animals. The PFC was dissected from all brains (by cutting out the frontal portion) which was then frozen in liquid nitrogen (−196°C) and subsequently stored in a container in the freezer (−80°C) until further processing.

### 2.2 Immunohistochemistry

The primary antibody used in the experiment was carefully chosen. The antibody against a neuron-specific nuclear protein (NeuN) is an excellent marker for neurons in the central and peripheral nervous systems in the embryo, juvenile, and adult individuals (Mullen et al., 1992). Every 25th frozen section was designated for routine immunoperoxidase labeling using DAB (Dako Liquid DAB + Substrate Chromogen System, K3468, Denmark) as a chromogen, which was described in detail in the previous paper (Kozłowska et al., 2019c). Selected sections were incubated overnight with a solution of primary antibodies directed toward NeuN (pan-neuronal marker; Anti-NeuN Antibody, clone A60, MAB377; Merck Millipore, Poland; working dilution 1:1000) and next incubated for about 1 h with the solution of secondary antibodies (working dilution 1:1000, ImmPRESS™ Universal Reagent). After that, sections were washed in PBS and incubated for 1 min with DAB substrate–chromogen solution prepared according to the manufacturer’s instructions. All stained tissues were rinsed in tap water, rehydrated through a graded alcohol solution, cleaned in xylene, and mounted in DPX (DPX Mountain for histology; 44,581, Sigma Aldrich, Germany). All stages of staining procedures were carried out at room temperature in humid chambers. The prepared sections were digitalized and archived using a PathScan Enabler IV Histology Slide Scanner (Meyer, USA).

### 2.3 Morphometric analysis of motor cortex

#### 2.3.1 Volumetric analyses

**Figure 1:**
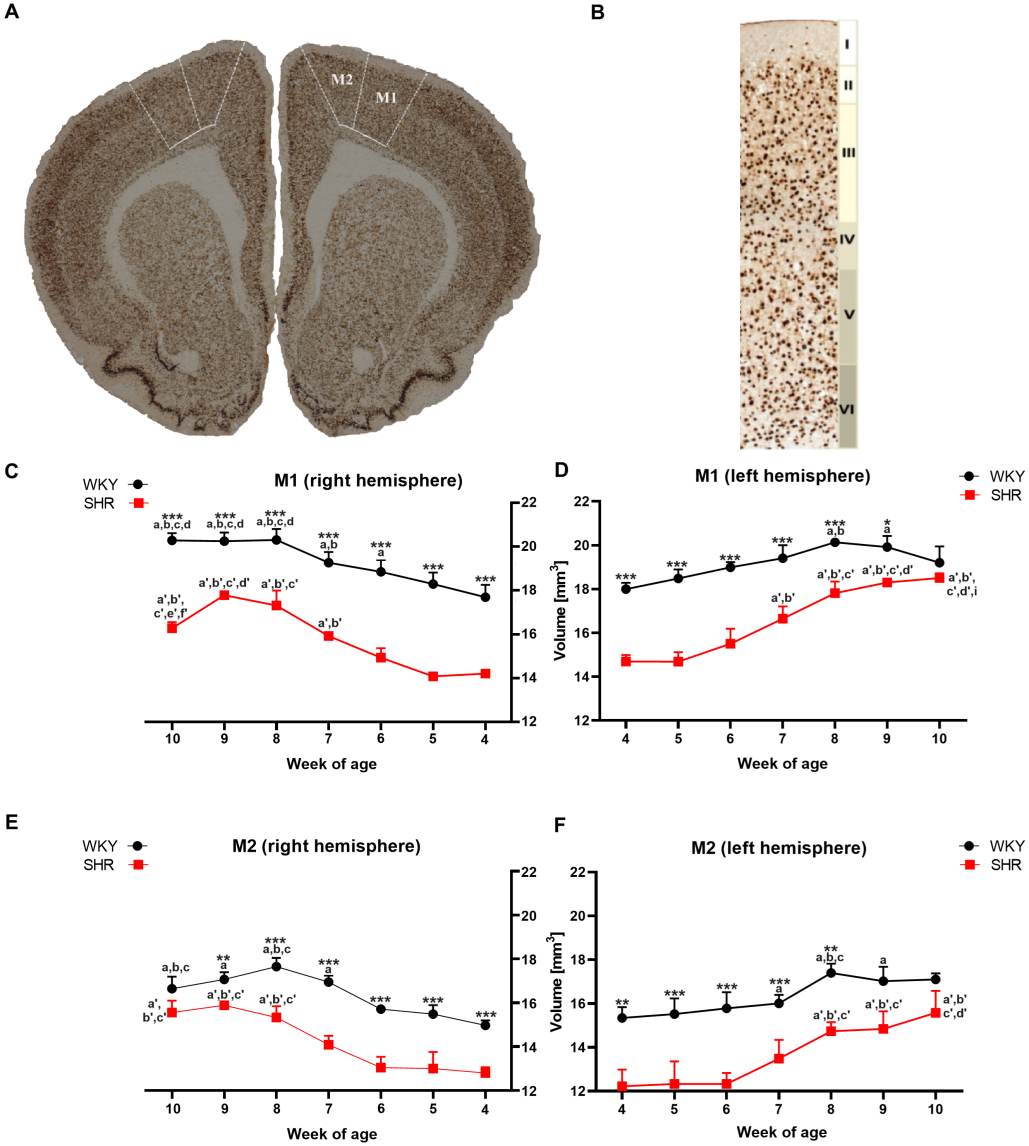
The figure shows **(A)** a coronal section of a rat brain (8-week-old WKY; magnification x 5.0) highlighting the primary (M1) and secondary (M2) motor cortex (bregma 2.28 mm; Paxinos and Watson, 2005). **(B)** NeuN-stained brain section illustrates cytoarchitectonic features in the M1 of 8-week-old WKYs. 10.0 x magnification, delineating cortical layers (I - molecular layer, II - external granular layer, III - external pyramidal layer, IV - internal granular layer, V - internal pyramidal layer, and VI - multiform layer). Moreover, volume comparisons of M1 **(C)** right hemisphere and **(D)** left hemisphere, and M2 **(E)** right hemisphere and **(F)** left hemisphere during their development (4–10 weeks) in SHRs and WKYs are shown (n = 5 or 6 for each strain at each age stage). Differences between strains: *p < 0.05, **p < 0.01, ***p < 0.001. Differences between rats dependent on age: a-f - indicate developmental differences (*p* < 0.05-*p* < 0.001) for WKYs strain; a’-f’ - indicate developmental differences (*p* < 0.05-*p* < 0.001) for SHRs strain; a, a’- 4 week vs. other weeks; b, b’ - 5 week vs. other weeks; c, c’ - 6 week vs. other weeks; d, d’ - 7 week vs. other weeks; e, e’ - 8 week vs. other weeks; f, f’ - 9 week vs. 10 week; i - indicate differences between hemispheres.

The boundaries of the distinct MC areas, including the M1 and M2, were precisely defined based on coordinates aligning with bregma 5.16 mm to −3.24 mm, referencing *The Rat Brain Atlas* by Paxinos and Watson (2005), sulcal landmarks in reference to previously published studies (Jeong et al., 2016; Barthas and Kwan, 2017) (Figures 1A). Briefly, volumetric measurements were performed on digitally archived coronal brain section images (5.0 x magnification, PathScan, Meyer, United States) using Fiji (Madison, United States) image analysis software (Schindelin et al., 2012). Sixty-eight (Koh et al., 2012) to eighty (Quinn, 2005) histology scan sections at the level of the M1/M2 were analyzed per animal. The mouse-driven cursor was used to outline the boundaries of M1 and M2 (right and left hemispheres) on each prepared digital slice. The total volumes of the individual M1 and M2 regions were calculated using the formula of DeVito et al. (1989):


$$V_{o}=\Sigma \;V_{n}$$

$V_{o}$ - total volume of the structure

$V_{n}$ - sub volumes through the structure

*V_n_ -* was calculated by multiplying 2-D contour area (depicting the boundary of the individual motor cortex region on the section) with the 250 μm space between sections

#### 2.3.2 The density quantification

Determining the cellular composition of specific motor cortex layers in SHR/NCrl and WKY/NCrl is crucial to understanding the participation of this structure in the pathophysiology of ADHD. In this experiment, we estimated the number of cell bodies in the M1 and M2 layers (I - molecular layer, II - external granular, III - external pyramidal, IV - internal granular, V - internal pyramidal, VI - multiform) (Alaverdashvili et al., 2014; Figure 1B) of both hemispheres from a single SHR/NCrl and WKY/NCrl. The motor cortex layers were distinguished by the cytoarchitectonic characteristics of this structure described in previous papers (Kriegstein and Dichter, 1983; Alaverdashvili et al., 2014). The analysis was performed on the sections from the beginning to the end of the brain at 250 μm intervals between sections. Every 4th section at the level of the M1/M2 were analyzed (17–20 sections per animal) at equidistant locations within this area. Quantitative analysis was performed manually on both the right and left sides with an Olympus BX51 microscope (magnification 40 x) equipped with a digital camera (Olympus, Germany) and a computer with Cell F software (Olympus, Germany). Neurons featuring a well-defined nucleus were counted in areas of individual motor cortex layers (M1/M2), precisely outlined using the mouse cursor. The Cell F software cell counter tool recorded mouse clicks on the neurons with distinct nuclei. Notably, nuclei positioned on the boundaries of the studied areas were not considered. The neuronal count for a specific layer of the structure underwent an initial averaging process across all examined brain sections. Subsequently, the averages for each developmental stage were computed and presented as the number of neurons per square millimeter (N/mm^2^).

### 2.4 Determination of immune, oxidative stress, and metabolic markers

Rationale for selecting immune, oxidative stress, and metabolic markers in the study can be found in Supplementary material 2.

#### 2.4.1 Preparation of test material

To ascertain the concentrations/activity of inflammatory, oxidative stress, and metabolic markers in the rat PFC, specific brain parts were isolated in accordance to *The Rat Brain Atlas* (Paxinos and Watson, 2005), followed by homogenization in RIPA buffer at 4°C. The homogenates were subsequently centrifuged at 30,000 × g for 1 h. Next, the resulting tissue supernatants were aliquoted and preserved at −80°C. These supernatants served as the basis for further measurements.

#### 2.4.2 Immunoenzymatic determination (ELISA) of the levels of immune markers such us cytokines: interleukin-1α (IL-1α), IL-1β, IL-6, serine/threonine-protein mammalian target of rapamycin (mTOR), RAC-alpha serine/threonine-protein kinase (AKT-1) and glucocorticoid receptor β (GCsRβ)

The concentrations of these markers in the PFC were determined using a commercial ELISA kits were used according to the manufacturer’s instructions (Supplementary material 2). The absorbance in this test plate was measured by plate reader TECAN Infinite M200 PRO (Austria) at the wavelength of *λ* = 450 nm. Results were presented per milligram of protein.

#### 2.4.3 Determination of oxidative stress markers

##### 2.4.3.1 The content of malondialdehyde (MDA) and sulfhydryl group (-SH)

MDA levels were assessed following the protocol outlined by Weitner et al. (2016), and the -SH content was determined using the Ellman method (Chan and Wasserman, 1993).

##### 2.4.3.2 The superoxide dismutase (SOD), peroxidase (POD), glutathione reductase (GSR) and glutathione S-transferase (GST) activity

For the evaluation of enzymatic activities, SOD and GSR activity was measured by the Tkachenko and Grudniewska method (Tkachenko et al., 2014), while GST activity was assessed through a spectrophotometric assay procedure (Habig et al., 1974). Additionally, POD activity was measured employing the kinetic method (Bovaird et al., 1982).

#### 2.4.4 Determination of metabolic markers

##### 2.4.4.1 The content of glucose (G), fructosamine (FrAm), iron (Fe) and lactic acid (LA)

The quantification of G, FrAm, Fe, and LA content was conducted employing specific commercial reagent sets as outlined in Supplementary material 2, following the manufacturer’s instructions.

##### 2.4.4.2 The activity of alanine transaminase (ALT), aspartate transaminase (AST) and lactate dehydrogenase (LDH)

The activity of ALT, AST and LDH was determined using appropriate commercial reagent sets according to manufacturer instructions. List of specific sets used to determine studied oxidative stress markers concentrations in rat PFC can be found in Supplementary material 2.

### 2.5 In-silico methods: protein–protein interaction analysis

Proteins are the fundamental structures of life and perform an essential role in most biological processes that are controlled through their mutual interactions (Lu et al., 2020). Protein–protein interactions (PPIs) in cells form a highly organized network called the ‘interactome’ (Venkatesan et al., 2009; Koh et al., 2012), which controls both physiological and pathophysiological processes, including signal transduction between compartments as well as growth, proliferation, cell differentiation and their apoptosis (Loregian and Palù, 2005; Arkin and Whitty, 2009; Nero et al., 2014). Given the recognition of abnormal PPI networks in the pathophysiology of neurodegenerative diseases (Blazer and Neubig, 2009), we decided to check interactions between studied immunological, oxidative stress and metabolic-related markers in the present study. For this purpose, we have used the online search tool STRING (Version: 11.5).

The *Rattus norvegicus* reference genome was used to generate the selected PPI networks using the STRING database with the cut-off criterion *p* < 0.05 and Markov Clustering (MCL) of nodes. Next, the results of these queries were filtered according to the required criteria: full STRING network, p-adjusted ≤0.05 (Benjamini–Hochberg correction), confidence equal to 0.400, the confidence of network edges, query protein only, and three groups of clusters. Finally, Gene Ontology Resource (GO, Gene Ontology Resource) enrichment analysis was applied to visualize and indicate corresponding biological functions for the markers under study.

### 2.6 Statistical analysis

Firstly, a preliminary analysis was conducted to assess normality using the Shapiro–Wilk test and homogeneity of variances using Levene’s test, confirming the assumptions necessary for subsequent analyses. Subsequently, a two-way ANOVA was performed with strain and time as independent factors. To ensure accuracy, reliability, and proper interpretation of the statistical results, Bonferroni post-hoc comparison tests were subsequently applied. Comparisons between hemispheres were made based on an ANOVA analysis that included the whole data sets (for both hemispheres). All statistical procedures were performed with the aid of GraphPad Prism 6 (GraphPad Software, La Jolla, CA, United States), and *p* < 0.05 was considered statistically significant. Detailed data regarding individual two-way ANOVA analyses are included in Supplementary material 3.

## 3 Results

### 3.1 Alterations in motor cortex

#### 3.1.1 Volumetric changes

##### 3.1.1.1 Primary motor cortex (M1)

A comparative analysis showed that the total volumes of M1 in almost all examined age stages of SHRs were significantly lower (*p* < 0.05 or *p* < 0.001) than in the WKYs (Figures 1C,D). Additionally, in the ADHD group, the total volumes of M1 in the right hemisphere (Figure 1C) at 10-weeks of life were significantly lower (p < 0.05) than in the left hemisphere (Figure 1D). In the control group, during weeks 6 to 8 of age a significant (p < 0.05–0.01) increase in the M1 volumes (right hemisphere) was observed, while in SHRs, this occurred only after the age of 7 weeks (*p* < 0.01). Interestingly, over 8 weeks of age, the volume of M1 in the WKYs was maintained at a similar level.

##### 3.1.1.2 Secondary motor cortex (M2)

Like M1, the total volumes of M2 in almost all examined age stages of SHRs (except 9 weeks in left hemisphere and 10 weeks in the right and left hemisphere) were significantly lower (*p* < 0.01 or *p* < 0.001) than in the WKYs (Figures 1E,F). However, unlike M1 in all age stages in the ADHD group, there were no statistically significant differences (*p* > 0.05) in the volume of M2 between the hemispheres. In the control and ADHD group, from 4 to 7 weeks of age, the total volume of M2 did not differ significantly. Whereas WKYs and SHRs in week 7/8 old, a significant increase (*p* < 0.001) in these volumes has been found (depending on the hemisphere), which remained unchanged to 10 weeks of age.

#### 3.1.2 The changes in the density of neurons in the particular layers of primary (M1) and secondary (M2) motor cortex

**Figure 2:**
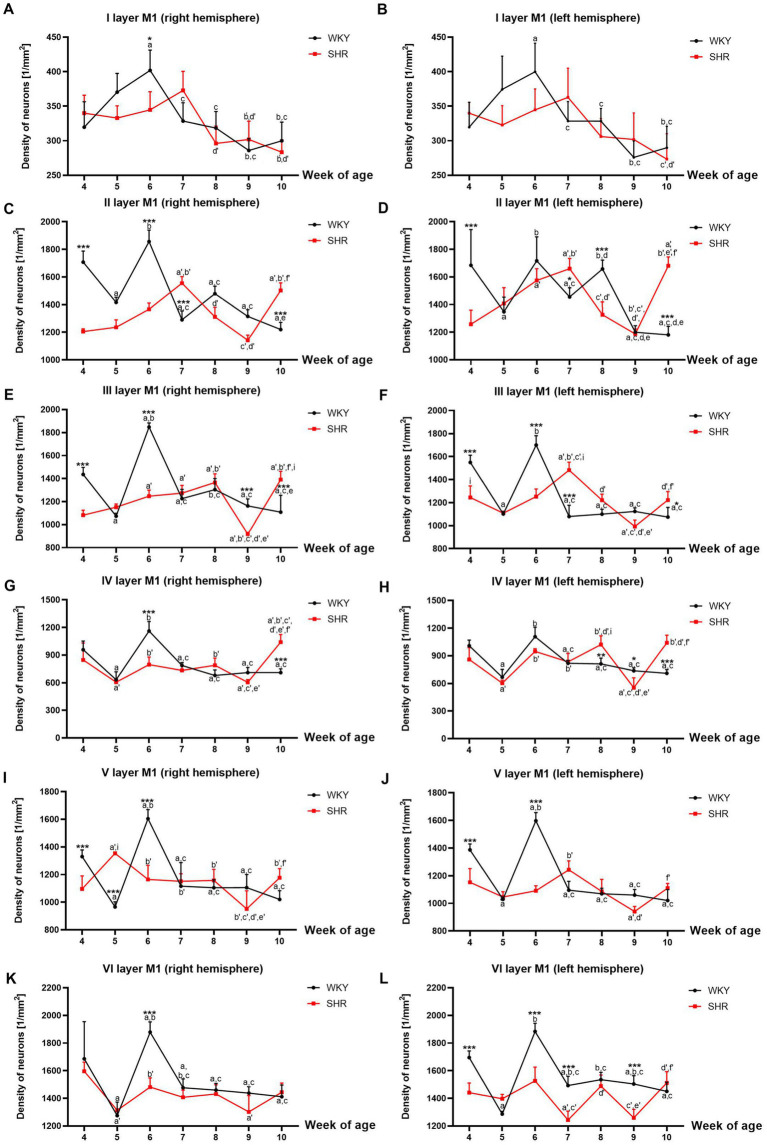
Comparison of neurons density [mm^2^] in particular layers of the primary motor cortex (M1): I layer in the right **(A)** and left **(B)** hemisphere; II layer in the right **(C)** and left **(D)** hemisphere; III layer in the right **(E)** and left **(F)** hemisphere; IV layer in the right **(G)** and left **(H)** hemisphere; V layer in the right **(I)** and left **(J)** hemisphere; VI layer in the right **(K)** and left **(L)** hemisphere during brain development (4–10 weeks) in SHRs and WKYs (n = 5 or 6 for each strain at each age). Differences between strains: ^*^p < 0.05, ^**^p < 0.01, ^***^p < 0.001. Differences between rats dependent on age: a-f - indicate developmental differences (*p* < 0.05-*p* < 0.001) for WKYs strain; a’-f’ - indicate developmental differences (*p* < 0.05-*p* < 0.001) for SHRs strain; a, a’- 4 week vs. other weeks; b, b’ – 5 week vs. other weeks; c, c’ - 6 week vs. other weeks; d, d’ - 7 week vs. other weeks; e, e’ - 8 week vs. other weeks; f, f’ - 9 week vs. 10 week; i - indicate differences between hemispheres.

**Figure 3:**
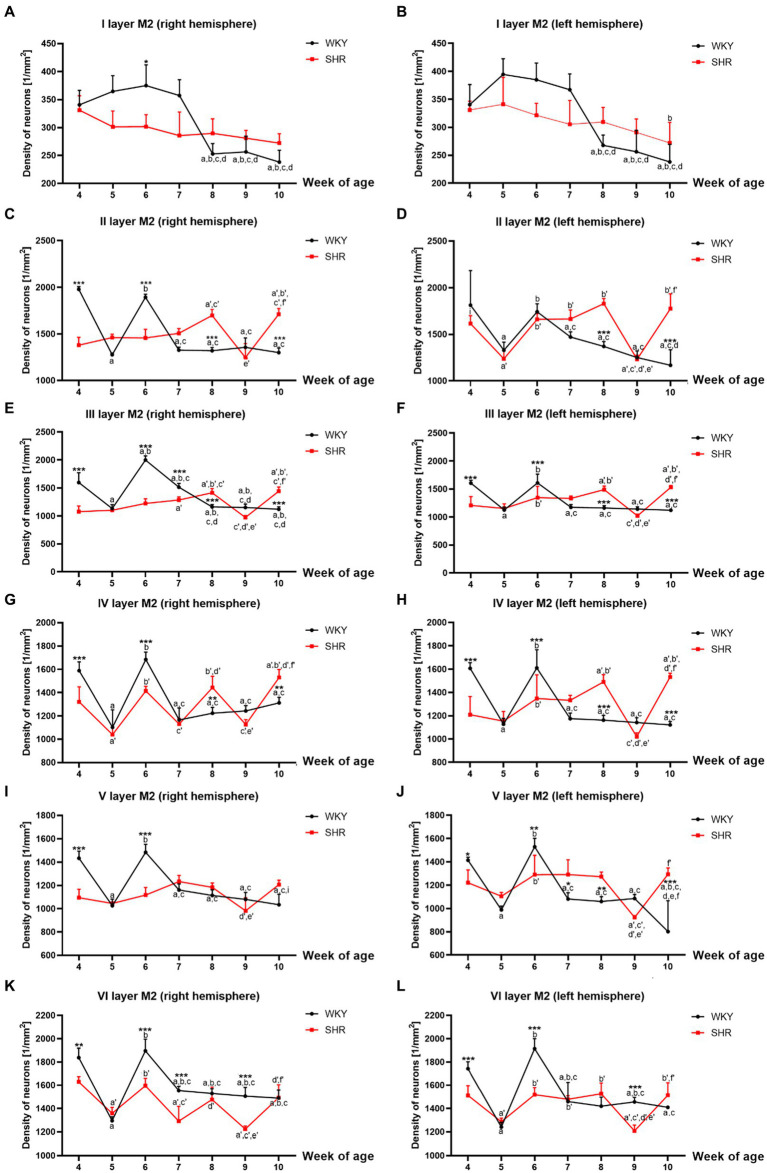
Comparison of neurons density [mm^2^] in particular layers of the secondary motor cortex (M2): I layer in the right **(A)** and left **(B)** hemisphere; II layer in the right **(C)** and left **(D)** hemisphere; III layer in the right **(E)** and left **(F)** hemisphere; IV layer in the right **(G)** and left **(H)** hemisphere; V layer in the right **(I)** and left **(J)** hemisphere; VI layer in the right **(K)** and left **(L)** hemisphere during brain development (4–10 weeks) in SHRs and WKYs (n = 5 or 6 for each strain at each age). Differences between strains: ^*^p < 0.05, ^**^p < 0.01, ^***^p < 0.001. Differences between rats dependent on age: a-f - indicate developmental differences (*p* < 0.05-*p* < 0.001) for WKYs strain; a’-f’ - indicate developmental differences (*p* < 0.05-*p* < 0.001) for SHRs strain; a, a’- 4 week vs. other weeks; b, b’ - 5 week vs. other weeks; c, c’ - 6 week vs. other weeks; d, d’ - 7 week vs. other weeks; e, e’ - 8 week vs. other weeks; f, f’ - 9 week vs. 10 week, i - indicate differences between hemispheres.

The mean neuron density of almost all studied layers in both motor cortex areas differed between SHRs and WKYs. Means for neuron density in each from all studied layers of M1 and M2 were listed in Figures 2, 3.

##### 3.1.2.1 Primary motor cortex (M1)

Mean neuron density in almost all studied layers of M1 with the exceptions of layers I, V and VI, was significantly higher in 7, 8 and/or 10-week-old SHRs (*p* < 0.05-*p* < 0.001) compared to their age-matched controls (Figures 2A–L). Whereas the density of neurons in these layers of 4, 6, and/or 9 weeks old was usually significantly lower (*p* < 0.05-*p* < 0.0001) than that observed in the WKYs (Figures 2A–L).

Neuron density changes in the SHRs had a unique pattern in all studied layers of M1. For example, the neurons number was relatively high in 7- and/or 10-week-old animals except for layers I (Figures 2A,B), V (Figures 2I,J) and VI (Figures 2K,L)]. Whereas, in 9-week-old SHRs, a significant drop (*p* < 0.05-*p* < 0.0001) in the neuron density was noted in layers: III (right hemisphere; Figure 2E), IV (left hemisphere; Figure 2H) and VI (left hemisphere; Figure 2L). It is worth adding that this pattern varied depending on the studied layer during the rest of the postnatal development.

Further analyses also revealed that the mean number of neurons differed significantly between the right and left hemispheres in the SHRs. For example, a higher density of these cells was seen for the first time in layers III in week 4 (*p* < 0.05) and 7 (*p* < 0.01; Figure 2F vs. Figure 2E) and layer IV in week 8 (*p* < 0.01; Figure 2H vs. Figure 2G) of the left hemisphere. In comparison, the right hemisphere had significantly higher neuronal density in layer III in week 10 (*p* < 0.01; Figure 2E vs. Figure 2F) and layer V in week 5 (*p* < 0.001; Figure 2I vs. Figure 2J) than on the left hemisphere.

Specific changes across postnatal development characterized neuron density in M1 of WKYs, as shown in Figures 2A–L. For example, the highest (p < 0.05–0.001, except for layer I) density of neurons in all studied layers was detected in 4- and/or 6-week-old animals. Between these developmental stages, in 5-week-old WKYs, the neuron density rapidly decreased (*p* < 0.0001). From 7 to 10 weeks, the density was maintained at a similar level (*p* > 0.05). Moreover, the distribution pattern of these cells was quite similar in the right and left hemispheres.

##### 3.1.2.2 Secondary motor cortex (M2)

Similarly to M1, almost all studied layers of M2 of 8 and/or 10-week-old SHRs had a significantly higher (*p* < 0.01–0.001) density of neurons than control animals except for I layer (right and left hemisphere; *p* > 0.05; Figures 3A,B), V layer (right hemisphere; p > 0.05; Figure 3I) and VI layer (right and left hemisphere; *p* > 0.05; Figures 3K,L). In contrast, in 4- and 6-week-old animals, the density was relatively low (*p* < 0.05–0.001) in most of the studied layers [with the exceptions of layer I - left hemisphere (Figures 3B), II - left hemisphere (Figure 3D)]. Additionally, a significant reduction in the neuronal density in 9-week-old SHRs was shown only in layer VI (*p* < 0.001; right and left hemisphere; Figures 3K,L).

All studied layers of M2 in SHRs displayed a unique cellular distribution pattern (Figures 3A–L). More precisely, neurons were more numerous (*p* < 0.05–0.01) in 8- and/or 10-week-old animals than in the remaining age stages studied. An increase in the density of neurons in 10-week-old SHRs observed in almost all layers of M2 (except for: I layer (Figures 3A,B), V layer (right hemisphere; Figure 3I) and VI layer (Figures 3K,L)) was always preceded by a decrease (*p* < 0.05–0.01) in average neuron density in 9-week of life. Whereas, at the other age stages of postnatal development of these animals, the pattern was changed according to the layer studied. Thus, in layers II (left hemisphere; Figure 3D), IV (right hemisphere; Figure 3G) and VI (right and left hemisphere Figures 3K,L) of 5-week-old SHRs a significant decrease (*p* < 0.01) in neuronal density was detected. Meanwhile, in 6 and/or 7-week-old animals, the density of neurons increased significantly (*p* < 0.05) in layer II (with the exception right hemisphere; Figure 3D), III (Figures 3E,F), IV (Figures 3G,H), V (except right hemisphere; Figure 3J), and VI (Figures 3K,L). Moreover, the comparative analysis of the right and left hemispheres of SHRs has revealed the differences in density between them. For example, in layer II in week 4, the neuron density of the left hemisphere (p < 0.05; Figure 3D) reached the highest value compared to the right hemisphere (Figure 3C).

The changes in neuronal density in WKYs across postnatal development were generally repeatable, as shown in Figures 3A–L. In a like manner the M1 and M2 of WKYs presented the highest (*p* < 0.01) neuronal density in weeks 4 and 6 of life. It is worth mentioning that between these developmental stages (in week 5), there was a significant drop (*p* < 0.05) in neuronal density. Moreover, in WKYs, from 7 to 10 weeks of life, the density was maintained at a similar level (*p* > 0.05).

Further analysis showed no hemispheric differences in WKYs during their postnatal development apart from layer V in week 10, where was observed the higher density of neurons in the right hemisphere (*p* < 0.01; Figure 3I) compared to the left hemisphere (Figure 3J).

### 3.2 Inflammatory markers

**Figure 4:**
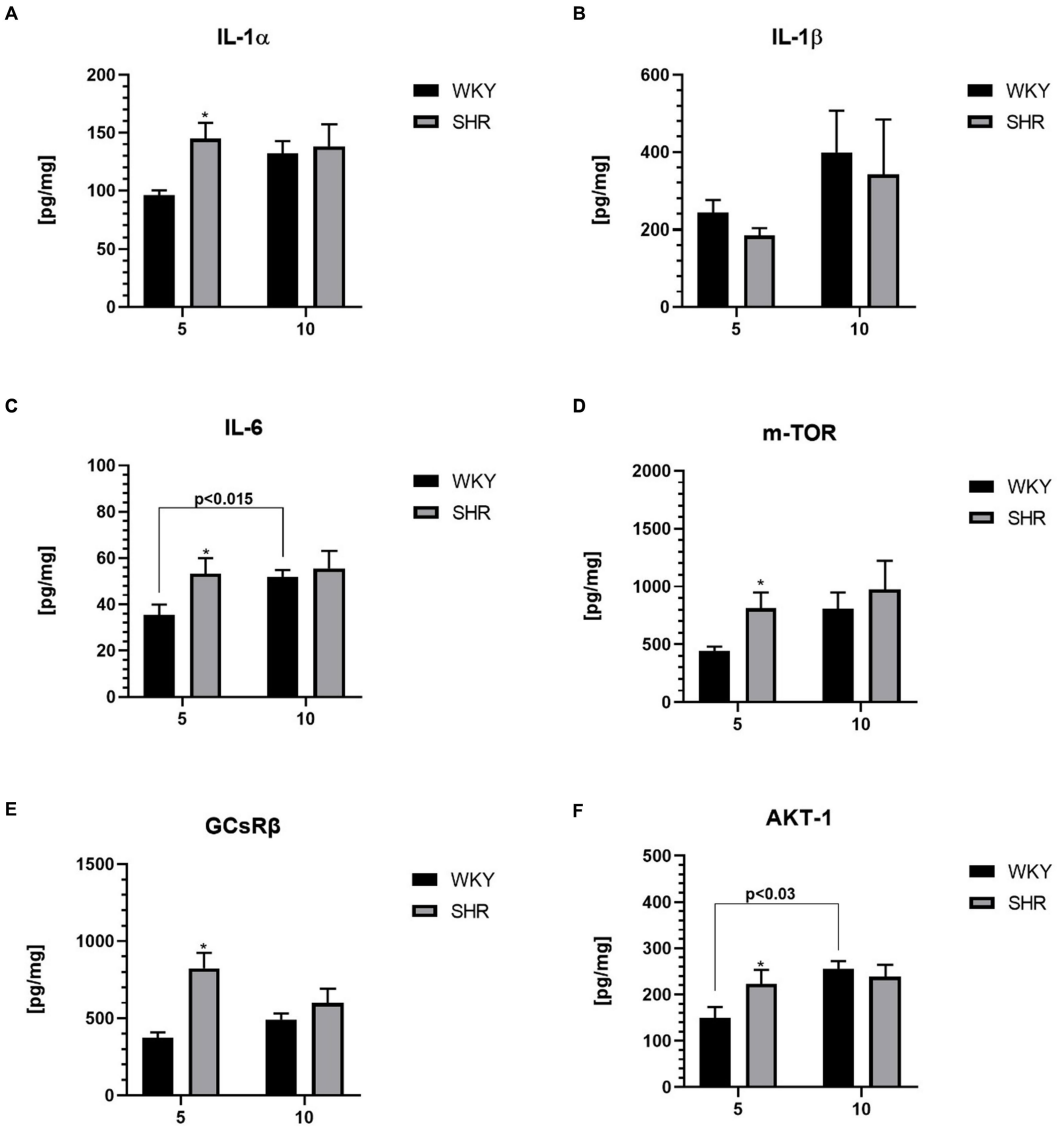
The levels of interleukin (IL)-1α **(A)**, IL-1β **(B)**, IL-6 **(C)**, serine/threonine-protein mammalian target of rapamycin (mTOR; **D**), glucocorticoid receptor (GCsRβ; **E**) and RAC-alpha serine/threonine-protein kinase (AKT-1; **F**) in PFC supernatants. The data are expressed as the mean ± SEM (*n* = 6/per group). The following statistical levels were applied: * indicates differences (*p* < 0.05) between SHRs and WKYs, *p* < 0.015, *p* < 0.03 indicate differences between the juvenile and maturing rats of the same strain.

The level of IL-1α (Figure 4A), IL-6 (Figure 4C), serine/threonine-protein mammalian target of rapamycin (mTOR; Figure 4D), glucocorticoid receptor β (GCsRβ; Figure 4E) and RAC-alpha serine/threonine-protein kinase (AKT-1; Figure 4F) were significantly higher (*p* < 0.05) in 5-week-old SHRs than in age-matched control. In contrast, IL-1β (Figure 4B) levels did not differ significantly between 5-week-old SHRs and WKYs (p > 0.05). Additional analyses showed that the level of IL-6 and AKT-1 were significantly elevated in 10-week-old WKYs compared to 5-week-old animals of the same strain (*p* < 0.015, *p* < 0.03; respectively). In comparison, the level of IL-1α, IL-1β, IL-6, m-TOR, GCsRβ and AKT-1 was similar in 10-week-old animals (p > 0.05).

### 3.3 Oxidative stress markers

**Figure 5:**
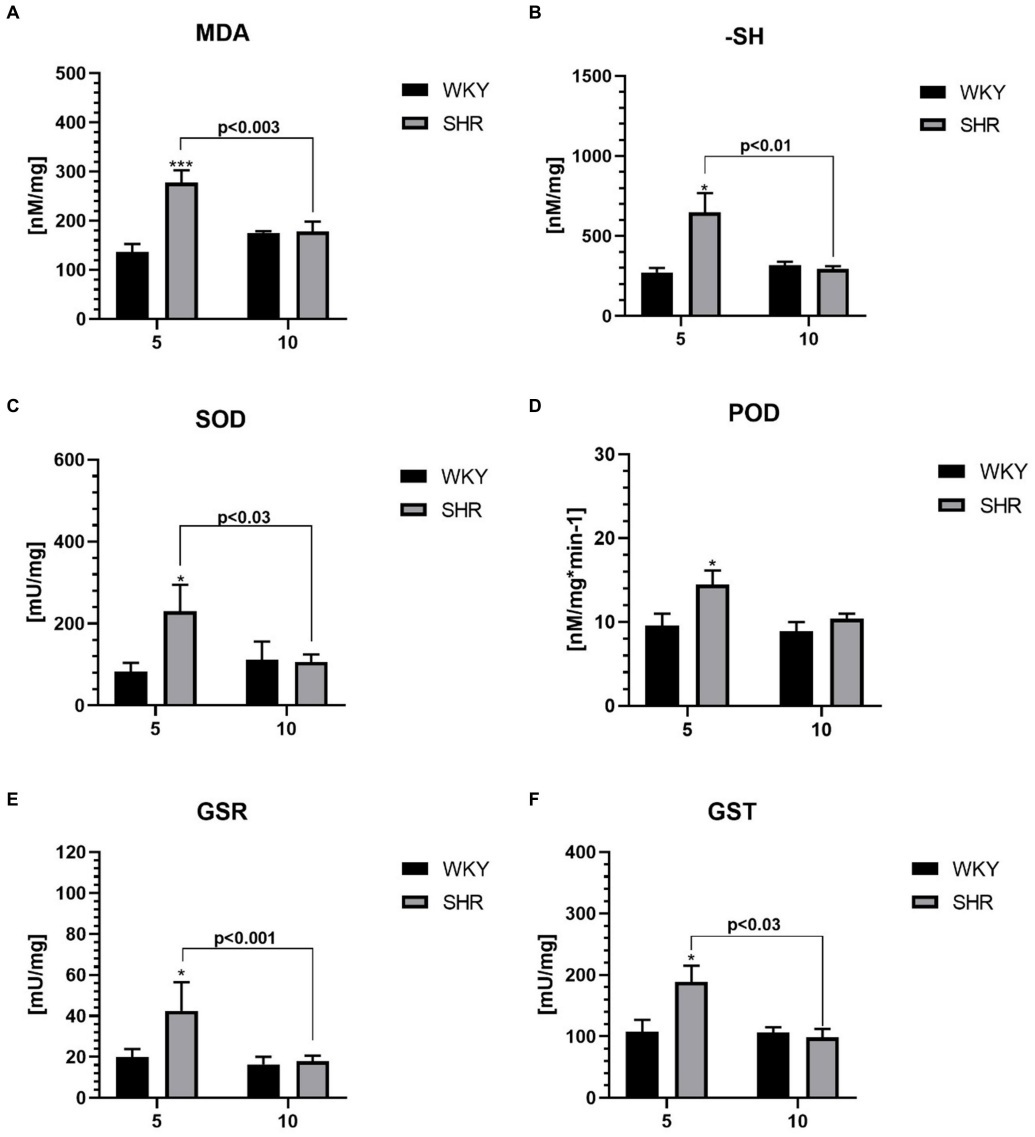
The levels of malondialdehyde (MDA) **(A)** and sulfhydryl group (-SH) **(B)**, superoxide dismutase (SOD) **(C)**, peroxidase (POD) **(D)**, glutathione reductase (GSR) **(E)** and glutathione S transferases (GST) **(F)** in PFC supernatants. The data are expressed as the mean ± SEM (*n* = 6 per group). The following statistical levels were applied: *, *** indicates differences (*p*<0.05, *p*<0.001) between SHRs and WKYs; *p*<0.03, *p*<0.01, *p*<0.003, *p*<0.001 - indicate differences between the juvenile and maturing rats of the same strain.

All studied oxidative stress markers were significantly higher in 5-week-old SHRs compared to age-matched control (Figures 5A–F). Moreover, the levels of MDA (Figure 5A), -SH (Figure 5B), SOD (Figure 5C), GSR (Figure 5E), and GST (Figure 5F) were significantly higher in 5-week SHRs compared to 10-week-old animals.

### 3.4 Metabolic markers

**Figure 6:**
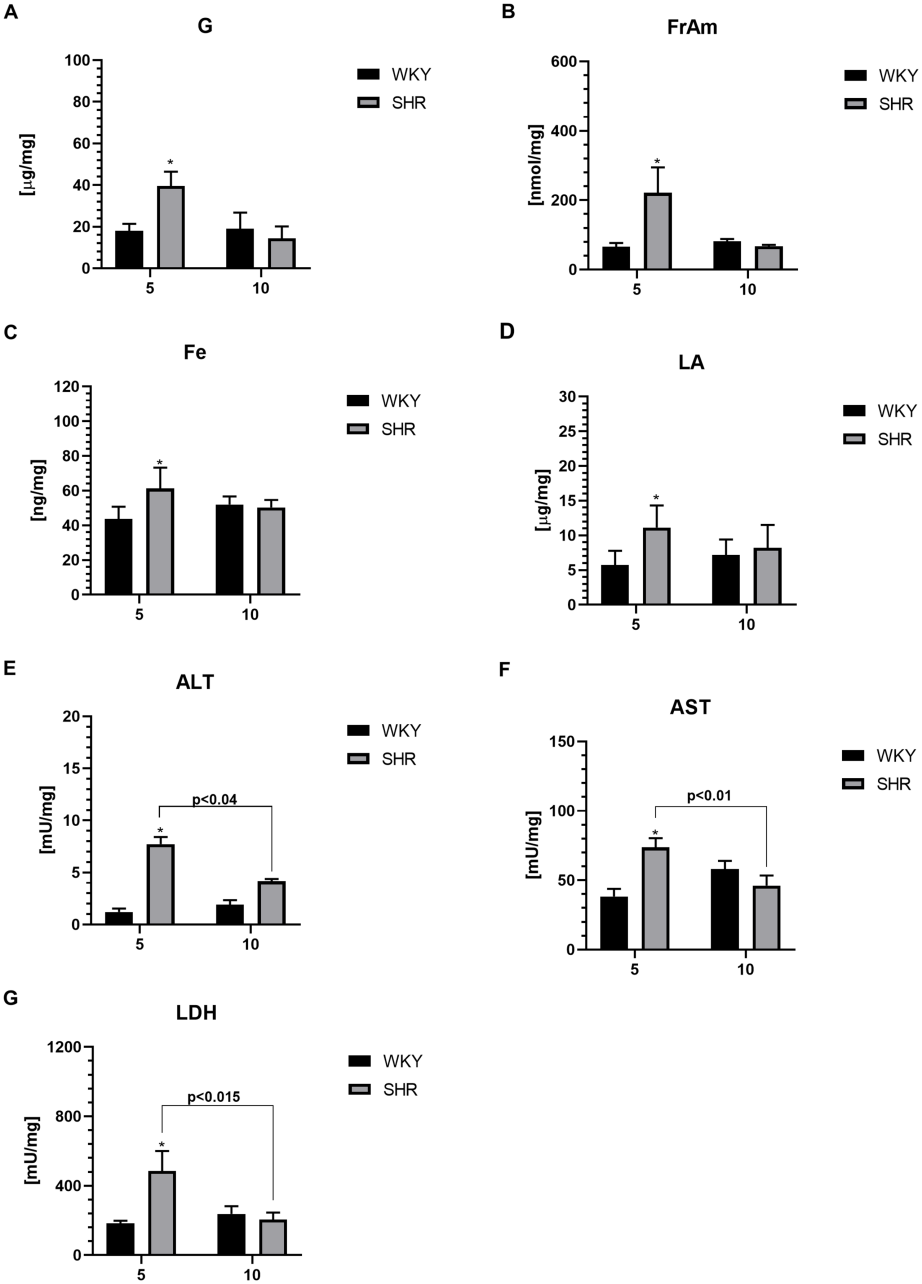
The levels of glucose (G) **(A)**, fructosamine (FrAm) **(B)**, iron (Fe) **(C)**, lactic acid (LA) **(D)**, alanine transaminase (ALT) **(E)**, aspartate transaminase (AST) **(F)** and lactate dehydrogenase (LDH) **(G)** in PFC supernatants. The data are expressed as the mean ± SEM (n = 6 per group). The following statistical levels were applied: *, ** indicates differences (*p* < 0.05, *p* < 0.01) between SHRs and WKYs *p* < 0.04, *p* < 0.015, *p* < 0.01 - indicate differences between the juvenile and maturing rats of the same strain.

The levels of glucose (G; Figure 6A), fructosamine (FrAm; Figure 6B), iron (Fe; Figure 6C), lactic acid (LA; Figure 6D), alanine transaminase (ALT; Figure 6E), aspartate transaminase (AST; Figure 6F) and lactate dehydrogenase (LDH; Figure 6G) in the PFC were significantly higher in 5-week-old SHRs than in aged-matched WKYs. Furthermore, the ALT, AST, and LDH level was also significantly higher in 5-week-old SHRs compared to 10-week-old rats of the same strain.

### 3.5 Protein–protein interaction (PPI)

**Figure 7:**
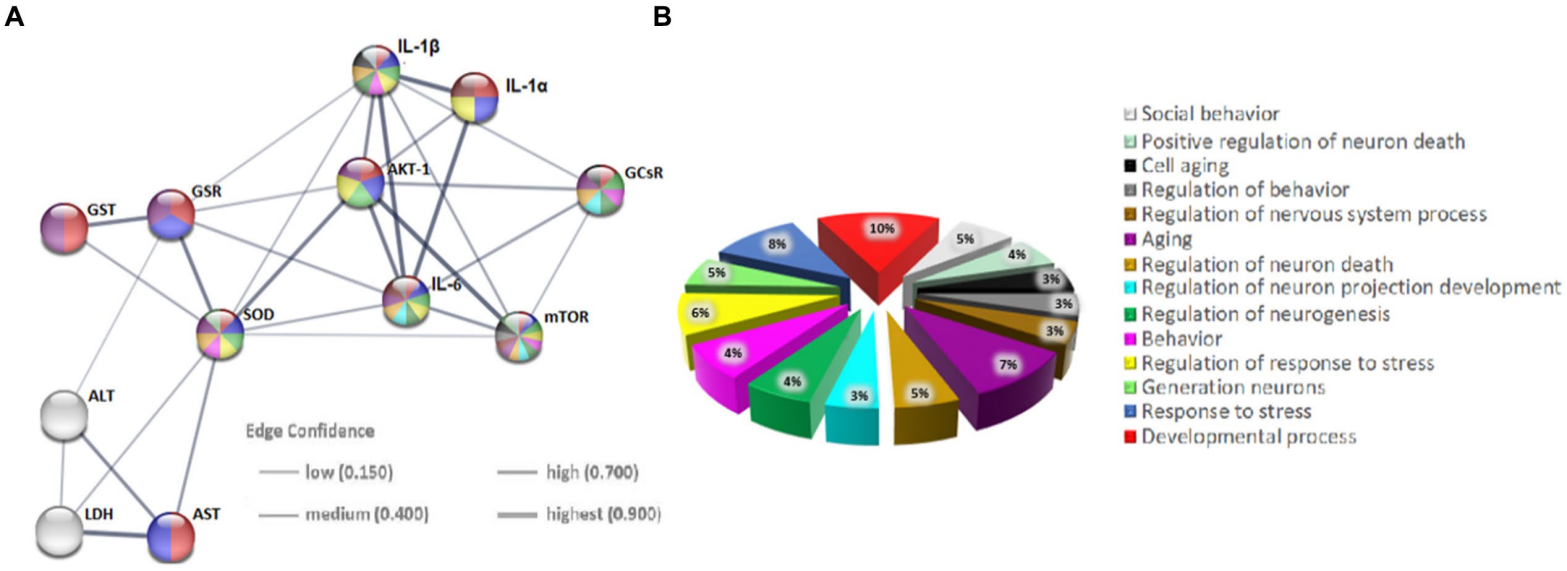
Protein–protein interaction (PPI) networks of potential targets in the STRING base (Rattus norvegicus). **(A)** The PPI network of 12 potential targets contains 12 nodes (proteins) and 7 edges (protein–protein associations). The thickness of the lines is in proportion to the confidence of the edges. **(B)** Pie chart of terms associated with neurodevelopment and behavior. Each color represents a specific biological process; note that the markers studied are implicated in these processes.

The evaluation of the protein–protein interactions (PPI) network analysis showed that it contained 12 nodes and 29 edges (Table 1); each node represented a target (immune, oxidative stress and metabolic markers), and each edge represented correlation evidence between 2 targets. The thickness of the edges representing the interacting proteins varies based on the combined confidence score for the protein association from the low 0.150 to the highest 0.900. All connected noodles indicated direct or indirect interactions between the 14 potential targets with 528 biological processes (BP) significantly enriched. The only 14 revalent BP connected with neurodevelopment and behavior were selected (*p* < 0.05; Supplementary Table S1; Figure 7; Supplementary material 4). The complete analysis found 100 reference publications (PubMed), 8 clusters of local networks (STRING) and 70 Reactome pathways.

**Table 1:** Network statistics and biological process (Gene Ontology) of the interactions.

Network statistics	
Number of nodes	12
Number of edges	29
Average node degree	4.83
Average. local clustering coefficient	0.702
Expected number of edges	7
PPI enrichment *p*-value	< 3.0 × 10^−10^

## 4 Discussion

The current investigation reveals significant delays in the maturation of the motor cortex (MC) regions and delayed neuronal differentiation within this structure in an animal model of ADHD development. Additionally, the observed morphometric abnormalities in this brain region were accompanied by disturbed homeostasis in immunological, oxidoreductive, and metabolic aspects within the prefrontal cortex (PFC) - a functionally and histologically distinct structure that collaborates with the MC to ensure effective motor control and coordination.

### 4.1 Motor cortex alternations

The present study reveals morphological abnormalities in the M1 and M2 regions of SHRs, with significant volume reductions observed at almost all ages. Detailed volumetric measurements of specific MC areas in SHRs have not yet been conducted. Similar changes were noted in our previous studies on other brain regions involved in motor activity, such as the PFC and striatum. Earlier reports also have shown reduced total volume of MC areas (equivalent to M1 and M2 in rodents) in children with ADHD (Castellanos et al., 2002; Kates et al., 2002; Mostofsky et al., 2002; Carmona et al., 2005; Batty et al., 2010) and adults (Proal et al., 2011).

It is noteworthy that Batty et al. (2010) reported a cortical thickness developmental trajectory in ADHD patients that appears to be similar to the observed in the presented study, considering age-related rat-human comparisons (Quinn, 2005). Additionally, our study finds more severe MC alternations in the right hemisphere, consistent with prior research brain structures linked to movement in SHRs (Hsu et al., 2010; Kozłowska et al., 2019b; Yang et al., 2021) and ADHD patients (Mostofsky et al., 2002; Almeida et al., 2010).

In addition to the observed changes in MC volume in SHRs, our study reveals a distinct pattern of neuron distribution in this brain area of SHRs compared to WKY over the studied lifespan. Specifically, the results demonstrate a decrease in neuronal density in most layers of M1 and M2 in juvenile SHRs (between 4 and 6 weeks of age). This indicates delayed maturation of these areas, consistent with observations previously reported in children with ADHD (Shaw et al., 2007, 2012). Comparing the presented results with existing data is challenging due to the limited literature on this topic. Confirmatory evidence showing reduced gray matter (GM) density in this brain region can be found in the study by Ashtari et al. (2005), which revealed lower Fractional Anisotropy (FA) in the MC of children with ADHD. Reduced neuronal density and deficits in GM in the MC area may cause complex disruptions in the anatomical organization large-scale brain networks (Griffiths et al., 2016; Wang et al., 2020), as reflected in studies of children with ADHD, who exhibited significantly reduced functional connectivity, particularly in prefrontal areas, including the MC (Gao et al., 2019; Wang et al., 2020).

This study demonstrates an increase in neuronal density during subsequent life stages of SHRs, culminating in significantly higher density in adult SHRs compared to age-matched WKYs. Interestingly, the observed increase in MC volume and the decrease in neuronal density in 9-week-old SHRs, may suggests brain ventricle enlargement, the phenomenon observed in other studies in SHRs of the same age (Lolansen et al., 2023). Furthermore, previous studies have demonstrated specific repair mechanisms activated after rapid brain ventricle enlargement (Tajima et al., 1979), potentially contributing to the observed increase in neuron density in 10-week-old animals. Specifically, it has been confirmed that intracranial pressure reduction led to rapid recovery of corticobasal pathways (an increase in FA), which was probably related to the restoration of axoplasmic flow (Eskandari et al., 2014; Jugé et al., 2016). Furthermore, it is important to emphasize that the dynamics of volumetric changes are not a direct reflection of the observed changes in neuronal density. This may stem from differences in volume measurement and neuronal density assessment methods. The first analysis encompassed both white matter (WM) and GM, whereas the second study focused solely on the GM area.

It is worth noting that the peak timing of significant morphometric abnormalities in the MC was identified during the prepubertal period, particularly between 4 and 6 weeks of age in SHRs. Since synaptic pruning processes occur during this period, it is probable that plasticity mechanisms are responsible for the developmental alterations in motor cortex associated with ADHD (Forero et al., 2009; Semple et al., 2013). This developmental window corresponds to approximately 6–10 years of age in humans (Quinn, 2005). Specifically, the most pronounced symptoms of hyperactivity and impulsivity occur in juvenile SHRs, followed by stabilization after the 10th week of age (Sagvolden et al., 2005; Hsieh and Yang, 2008). Similarly, this time frame also correlates with the peak severity of hyperactivity symptoms in ADHD children (Bitsko et al., 2022).

One intriguing finding is the notable drop in neuronal density observed in 5-week-old WKYs. This may be due to the fact that rat brain development is known to show intense alterations in the cellular composition of the brain, the growth of which is modulated by different combinations of programmed cell proliferation and apoptosis during the early postnatal stages (Bandeira et al., 2009). This phenomenon may be attributed to reorganizational and adaptive changes in the central nervous system associated with the transition from childhood to adulthood. Specifically, puberty, occurring around 28–42 days after birth in rats, is characterized by phases of both reduced and increased neuroplasticity in subcortical and cortical regions (Spear, 2000). The dynamics of these changes are determined by elevated levels of gonadal steroid hormones (estradiol, testosterone - T, dehydroepiandrosterone) appearing during this period (Laube et al., 2020). Particularly, estradiol exposure initiating early maturation may lead to decreased brain plasticity (Spear, 2000). Additionally, this hormones have been shown to contribute to reduced GM volume (Kolb and Gibb, 2011). Furthermore, T has been associated with increased WM volume, which may explain the lack of noticeable declines in MC volume during adolescence presented in our results (Willing and Juraska, 2015).

Finally, it is worth noting that the brain regions studied directly correspond to regions delineated in *The Rat Brain Atlas* (Paxinos and Watson, 2005). This aspect constitutes a significant advantage compared to structural MRI studies, where image contrast may not be sufficiently high to detect boundaries between different rat brain compartments (Jing et al., 2018).

### 4.2 Inflammatory markers

The present results underscore a noteworthy elevation in the levels of nearly all assessed inflammatory markers in the prefrontal cortex (PFC) of 5-week-old SHRs compared to age-matched WKYs, except for IL-1β. Similarly, elevated levels of IL-6 in the PFC have been reported in juvenile SHRs (Song et al., 2021). Unfortunately, current literature shows a significant lack of data regarding IL-1β content in brain tissue of both SHRs and ADHD patients. However, IL-1α and IL-1β, belonging to the same IL-1 family, share the same receptor and have strong pro-inflammatory properties (Sobowale et al., 2016). Interestingly, our current study indicates unchanged PFC content of IL-1β in 5-week-old SHRs, while in our prior study, serum this cytokine levels increased (Kozłowska et al., 2019b). Similarly, elevated serum levels of IL-1β have previously been observed in ADHD children but not adults (Xiang, 2012). This phenomenon, i.e., the lack of differences in this cytokine content in PFC between SHRs and WKYs in both age groups, might be attributed to differential bioavailability of IL-1β and IL-1α in various tissues (Eislmayr et al., n.d.). Notably, our study reveals elevated IL-6 levels in young SHRs, which have previously been reported in serum of SHRs and ADHD children (Oades et al., 2010; Kozłowska et al., 2019b). Although the mechanisms of IL-6 action are not fully elucidated, research suggests that the administration of this cytokine in rodents results in a reduction of brain DA levels (Zalcman et al., 1994).

Additionally, we observed elevated levels of PI3K/AKT/mTOR pathway enzymes, namely mTOR and AKT-1, in juvenile SHRs. This pathway has been implicated in the pathophysiology of ADHD, as it is fundamental for regulating neuron proliferation, maturation, and integration into mature brain circuits (Sánchez-Alegría et al., 2018; Yde Ohki et al., 2020). The increased levels of mTOR and AKT-1 in juvenile SHRs may indicate dysregulation of this signaling pathway. This have been associated with negative effects such as oxidative imbalance, membrane depolarization, mitochondrial damage, increased inflammation, and energy disruption (Seitz et al., 2013; Liu et al., 2014; Chu et al., 2021). In these conditions, microglia adopt a neurotoxic phenotype and can accelerate neuronal damage (Magdalon et al., 2017; Chu et al., 2021), which may explain the reduced neuron density observed in juvenile SHRs in this study. Additionally, evidence suggests that several mutations in proteins involved in this pathway are linked to ADHD symptoms (Lee, 2015; Karalis and Bateup, 2021). Notably, inflammation-activated microglia release regulatory proteins and pro-inflammatory cytokines, which have been shown to impair glucocorticosteroid receptor (GCsRβ) activity (Pace and Miller, 2009), increasing glucocorticosteroid resistance.

In the conducted study, we observed significantly elevated levels of GCsRβ receptors in the PFC of juvenile individuals with ADHD, consistent with previous reports (van der Meer et al., 2016; Wu et al., 2017). Additionally, our findings are supported by the study of van der Meer et al. (2016), which demonstrated overexpression of the NR3C1 9β gene encoding GCsRβ in children with ADHD (Derijk et al., 2001). Dysregulation of the hypothalamic–pituitary–adrenal (HPA) axis in psychiatric disorders (Misiak et al., 2020) is often linked to attention, arousal, perception, memory, and emotional processing impairments commonly attributed to ADHD (Erickson et al., 2003; Pace and Miller, 2009). Moreover, recent data highlight a significant association between GCsRβ overexpression and GM volume reduction in the cerebral cortex of children with ADHD (van der Meer et al., 2016), which may further explain the reduced neuron density observed in juvenile SHRs.

Interestingly, no differences were observed in the levels of the examined inflammatory markers between adult SHRs and age-matched WKY rats, which is consistent with previous reports. (Leffa et al., 2017). This discrepancy might be attributed to significantly elevated serum and adrenal cortisol and corticosterone levels observed in adult SHRs (Kozłowska et al., 2019b,c). As it is known, glucocorticoids are adaptive hormones with immunosuppressive properties, reducing pro-inflammatory cytokine levels (Coutinho and Chapman, 2011). Moreover, it is worth noting that juvenile SHRs showed high levels of IL-1α and IL-6, which correlate with markers of metabolic and oxidative stress. However, while metabolic and oxidative stress normalize with age, inflammatory markers remain elevated. This suggests that immunological abnormalities during this critical period (adolescence) may be a predominant mechanism in the pathogenesis of ADHD, rather than oxidative and metabolic changes (Dunn et al., 2019).

Concurrently, we observed a significant increase in IL-6 and AKT-1 levels in 10-week-old WKYs compared to their 5-week-old counterparts, consistent with previous findings (Chen et al., 2019; Kozłowska et al., 2019a; Porcher et al., 2021). Cytokine upregulation may result from brain aging processes, which have been associated with the upregulation of numerous cytokines, including those mentioned above (Chen et al., 2019; Porcher et al., 2021).

### 4.3 Oxidative stress markers

The existing body of literature suggests a connection between the pathophysiology of ADHD and disruptions in oxidative stress balance (Dunn et al., 2019; Corona, 2020). However, the definitive causal relationship has not yet been determined. Notably, our current findings reveal a significant elevation in the levels of MDA, a biomarker for oxidative stress and a byproduct of lipid peroxidation (Bulut et al., 2007), in the PFC of juvenile SHRs. These is in line with previous studies reporting heightened MDA levels in the PFC of SHRs (Coelho-Santos et al., 2018) and in the serum of children diagnosed with ADHD (Verlaet et al., 2019). Nevertheless, conflicting reports exist; for instance, Oztop et al. (2012) reported lower plasma MDA levels in children with ADHD. Such disparities may stem from variations in biomarker accessibility within specific tissues and differences in the sensitivity and specificity of assay methods (Taş et al., 2014; Carraro et al., 2018). Consequently, comparing our findings becomes particularly challenging.

Nonetheless, it has been shown that oxidative stress in the PFC of juvenile SHRs may impair dopaminergic neurotransmission (Meiser et al., 2013) and thereby contribute to the increased hyperactivity and reactivity observed in SHRs (Tsai et al., 2017) as well as in patients with ADHD (Blum et al., 2008). Furthermore, oxidative stress might impact alterations in neuronal cell morphology, differentiation, migration, and plasticity (Ceylan et al., 2010; Verlaet et al., 2019), which could influence the observed reduction in neuron density in juvenile SHRs. Our study also unveiled a significant increase in the activity of antioxidant enzymes, such as superoxide dismutase (SOD) and peroxidase (POD), in the PFC of 5-week-old SHRs. To date, only reports indicating no significant differences in SOD levels in the blood of pediatric patients with ADHD have been published (Ceylan et al., 2010). The divergence in SOD activity between our findings and those of Ceylan et al. (2010) may be attributed to variances in its activity between serum and brain tissue, as previously observed in rats (Boriskin et al., 2019).

Importantly, recent findings have demonstrated a notable increase in the total antioxidant status (TAS) in children and adolescents with ADHD (Selek et al., 2012). Our study suggests an intensified oxidative stress in young SHRs, potentially triggering compensatory mechanisms, as indicated by the concurrent increase in oxidants and antioxidants in the PFC (Lee et al., 2020). Additional evidence supporting the activation of adaptive responses to oxidative stress includes the elevation of sulfhydryl groups (-SH), glutathione reductase (GSR), and glutathione S-transferase (GST) levels in the PFC of juvenile SHRs. Unfortunately, detailed data on these antioxidant levels in the PFC of SHRs and/or ADHD individuals are lacking. For -SH, reports only indicate higher levels in the saliva and plasma of children with ADHD (Archana et al., 2012; Oztop et al., 2012) and in the spleen of juvenile SHRs (Kozłowska et al., 2019b). Nevertheless, these results exhibit occasional inconsistencies. Notably, Öğütlü et al. (Öğütlü et al., 2020) have documented decreased serum -SH levels in children with ADHD compared to unaffected individuals, introducing a note of contradiction to the existing body of evidence. This inconsistency may be attributed to the varying availability of these groups in specific tissues and/or species-specific differences. Previous investigations underscore the critical role of dynamic thiol/disulfide homeostasis in antioxidant mechanisms, apoptosis, detoxification, cellular signal transduction, and the regulation of enzyme and transcription factor activity (Baba and Bhatnagar, 2018). Elevated -SH levels have been linked to the pathogenesis of several neuronal diseases, including Parkinson’s, Alzheimer’s disease, Friedreich’s ataxia, multiple sclerosis, and amyotrophic lateral sclerosis (McBean et al., 2017). The observed increase in oxidative stress in juvenile SHRs induced an elevation in the levels of glutathione reductase (GSR) and glutathione S-transferase (GST). These enzymes are functionally linked and participate in the detoxification of aldehyde compounds like MDA (Vašková et al., 2023). Regarding GST, our results are in line with reports of significantly elevated enzyme levels in the plasma of children with ADHD (Çelik et al., 2013), while conflicting findings have been reported by Ceylan et al. (2012), potentially due to methodological differences and tissue specificity (Knight et al., 2007). It is worth mentioning that an increase in GSR activity in response to oxidative stress has been observed in the cerebral cortex of rodents (Carissimi et al., 1999; Erejuwa et al., 2012). Interestingly, our findings indicate a decline in the levels of all oxidative markers with age in SHRs, reaching statistically similar values to WKYs at 10 weeks of age, in accordance with prior evidence (Leffa et al., 2017). This age-related decrease may stem from compensatory-adaptive mechanisms against oxidative stress (Karababa et al., 2017).

It is important to highlight that the PFC stands out as a region of the brain exceptionally vulnerable to damage induced by oxidative stress (Schmitz et al., 2012). Important arguments supporting our hypothesis that inflammation and oxidative stress observed in the PFC of SHRs are related to ADHD include literature evidence showing that the administration of anti-inflammatory and antioxidant substances resulted in a reduction in ADHD symptoms in human patients (Richardson and Puri, 2002; Manor et al., 2012; Perera et al., 2012) as well as in SHRs (Song et al., 2021).

### 4.4 Metabolism markers

Spectrophotometric analysis revealed a significant increase in glucose (G) and fructosamine (FrAm) levels in the PFC of 5-week-old SHR rats. These is in line with previous studies showing reduced G metabolism in the brains of children with ADHD (Ernst et al., 1994). Disrupted G homeostasis in the PFC is directly linked to elevated FrAm level - a glycoprotein formed under hyperglycemic conditions through non-enzymatic protein glycation (Welsh et al., 2016). Elevated FrAm levels have also been observed in various conditions often comorbid with ADHD, such as psychosis, bipolar disorder, and depression (Garcia-Rizo et al., 2016). The increased G and FrAm content in the PFC of young SHR rats in our study may result from an imbalance in oxidative stress and the activation of inflammatory cascades. These conditions can disrupt mitochondrial membrane ion polarization, leading to impaired mitochondrial function, including pyruvate transport and metabolism - a product of the glycolysis pathway related to G (Han et al., 2001). Disrupted transport of this product into mitochondria leads to insufficient energy production needed for these organelles’ survival. Under these conditions, mitochondria may induce apoptosis, potentially leading to neuronal death, neurodegeneration, and GM deficits (Stefano et al., 2016; Liu et al., 2020). Moreover, increased oxidative stress is associated with a shift from mitochondrial aerobic metabolism to anaerobic metabolism, which involves cytoplasmic biochemical pathways (Verma et al., 2016; Ježek et al., 2023). This is a feature of hypoxia, where enhanced anaerobic metabolism leads to increased pyruvate and lactic acid (LA) concentrations (Magistretti and Allaman, 2018). Additionally, this metabolic shift is accompanied by increased lactate dehydrogenase (LDH) activity, facilitating the conversion of pyruvate to LA. Our study showed elevated LDH and LA levels in the PFC of 5-week-old SHR rats compared to the control group. These findings are consistent with previous studies showing increased expression of the LA transporter MCT1 in brain microvessels and enhanced LA transport from the periphery to the brain across the blood–brain barrier in young SHR rats (Medin et al., 2019). On one hand, LA serves as a primary energy substrate when G metabolism is disrupted (Xue et al., 2022). On the other hand, elevated LA levels in the PFC may increase the formation of lactic acid complexes with calcium, contributing to the downregulation of the GABAergic system, which may lead to symptoms of hyperactivity and impulsivity observed in ADHD (Bollmann et al., 2015; Chen et al., 2022). The precise roles of LDH and LA in brain function in ADHD remain the subject of ongoing discussion. Nevertheless, the increased production of LA and enhanced LDH activity in our study are consistent with previous reports of mitochondrial dysfunction and impaired neuronal energy metabolism in the pathogenesis of ADHD (Verma et al., 2016; Öğütlü et al., 2020). It is well known that mitochondrial dysfunction causes a loss of cellular integrity and triggers apoptosis/necrosis (Cowan et al., 2019). This can be assessed through alanine aminotransferase (ALT) and aspartate aminotransferase (AST) activity, commonly used as markers of necrosis, cellular damage, and mitochondrial dysfunction (Rossignol and Frye, 2012; McGill, 2016). Our study reveals significantly elevated ALT and AST levels in the PFC of young SHR rats compared to their control peers, consistent with previous observations of elevated serum ALT levels in these animals (Gao et al., 2019). Although no studies have directly reported these marker levels in ADHD patients. Skalny et al. (2021) found elevated serum alanine and asparagine levels in children with ADHD, which may indicate increased AST and ALT activity (Skalny et al., 2021). It is worth noting, that iron (Fe) metabolism is closely linked to mitochondrial function and is crucial for cellular metabolism. Briefly, extracellular Fe is taken up by cells and transported to mitochondria, where it is used to synthesize cofactors essential for enzymes involved in redox reactions, DNA synthesis and repair, and various cellular processes (Horowitz and Greenamyre, 2010). In our study, we observed significantly elevated Fe levels in the PFC of young SHR rats compared to their control peers. The elevated level of this element may suggest tissue damage within the PFC, consistent with a recent study showing increased Fe and ferritin levels following brain damage induced by hypertension or ischemia in SHR rats (Yang et al., 1993; García-Yébenes et al., 2012). Intriguingly, previous studies investigating the relationship between serum Fe levels and ADHD in humans have produced inconclusive results. Chen et al. (2004) observed significantly elevated serum Fe levels in children with ADHD, while meta-analyze suggested no differences (Chen et al., 2004). Discrepancies in human studies may result from differences in Fe intake and other factors significantly affecting this element availability in children’s diets (Collings et al., 2013). Importantly, differences in Fe levels in pathologically altered tissues may be greater than in peripheral tissues such as blood, plasma, and serum (Navas and Córdova, 2000). This study suggests that elevated Fe levels in the PFC of young SHR rats may contribute to and/or be a marker of neurodegenerative changes in brain area (Ferreira et al., 2019). This hypothesis is supported by evidence indicating that excess Fe in the brain can secondarily induce oxidative stress, lipid peroxidation, blood–brain barrier destruction, and neuronal death (Zhang et al., 2014; Cheng et al., 2022).

Lastly, it’s noteworthy that our study did not reveal differences in the levels of metabolic markers in adult SHRs compared to controls. This observation may be a consequence of the suppression of inflammation and oxidative stress in adult animals, attributed to compensatory mechanisms as previously discussed (Karababa et al., 2017; Kozłowska et al., 2019b).

### 4.5 Protein–protein interaction analysis

The additional in-silico analysis using STRING software provides strong support for our hypothesis. Namely, it confirmed a significant association between the studied immunological, oxidative stress and metabolic markers on neurodevelopmental and behavioral processes. More specifically, the string database enabled the setting up of a protein–protein interaction (PPI) network for all protein markers analyzed in the present study exhibiting strong interactions between them. In addition, the use of GO molecular function enrichment analysis allowed the detection of fourteen terms (biological processes) involved in the neurodevelopment and behavior of *Rattus norvegicus* in general, mediated by the protein markers studied. It is important to note that biochemical pathways, along with the enzymes that comprise them, are typically conserved and can induce similar reactions in different cells within the same tissue or organ. Notably, the PPI analysis presented here provides an important jumping-off point for further research into the interactions between immune, oxidoreductive, and metabolic factors in the pathogenesis of ADHD.

In conclusion, *in silico* analysis using STRING software strongly supports the hypothesis of significant links between immunological, oxidative stress and metabolic markers affecting neurodevelopment and behaviour. The identified network of protein–protein interactions and the resulting biological functions may point to future investigation directions for finding in the context of ADHD etiopathogenesis.

## 5 Limitations of a study

Although SHRs are widely accepted as a model for ADHD, it is essential to acknowledge that results from animal models do not always directly translate to the pathophysiology of this disorder in humans. For example, discussions are ongoing about the similarities and differences between the motor areas of the rat’s primary (M1) and secondary (M2) motor cortex (MC) and their primate counterparts. The M1 and M2 areas in rodents, along with the MC in primates, share the overarching function of controlling voluntary movements. Additionally, both rats and primates have a MC organized into layers of cells (Ferreira et al., 2019; Muñoz-Castañeda et al., 2021). Furthermore, there are homologous regions in the MC of rats and primates. For instance, the forelimb area in rats corresponds to the representation of the arm and hand in primates (Zhang et al., 2014; Cheng et al., 2022). In both primates and rodents, lesions in the M1 or prefrontal cortex (PFC) result in a consistent set of symptoms that show remarkable similarities between the two species (Park et al., 2012). While the aforementioned studies are based on significant evidence indicating similarities in the MC of rats and primates, it is important to note that primates have a more complex motor cortex with distinct areas such as the M1, supplementary motor area and premotor cortex, each contributing to different aspects of motor control and planning. Therefore, direct comparisons between the results obtained in SHRs and the pathophysiology of ADHD in humans should be made carefully.

It should also be mentioned that the studies presented focus on SHRs aged 4 to 10 weeks to investigate changes in the MC and PFC in the course of ADHD. Although this age range covers key developmental stages in the pathophysiology of this neurodevelopmental disorder, it is crucial to consider whether hypertension, a characteristic feature of SHRs, might influence the results. According to the literature, early development of hypertension in SHRs begins after 7 weeks of age (Park et al., 2012). In this study, most neuroanatomical and biochemical changes were observed between 4 and 6 weeks, allowing us to disregard the influence of hypertension on the obtained results.

## 6 Conclusion

The data presented in this manuscript shed light on significant delays in the maturation of MC areas and disturbed neuronal differentiation in this structure in an animal model of ADHD development. These changes in MC, along with disrupted homeostasis in immunological, redox, and metabolic aspects within the PFC. As is well known, the PFC interacts closely with M2 and M1 through both input and output neuronal connections, which are crucial for the coordination and execution of complex cognitive and motor functions. Therefore, inflammation, oxidative stress and altered metabolism in juveniles of an animal model of ADHD in the PFC may affect dysfunctions in neurotransmission between these areas of the cortex, influence morphometric alterations in these structures, and consequently exacerbate symptoms of hyperactivity and impulsivity. These factors highlight the complex interaction of various alterations underlying the pathophysiology of ADHD.

Previous reports also have shown reduced MC volume and altered neuron density in children and adults with ADHD, suggesting a common underlying mechanism. To elucidate the causes of these changes, the study delves into markers of inflammation, oxidative stress, and metabolism in the PFC. The observed increase in levels of these markers in young SHRs may contribute to gray matter deficits, disturbances in dopaminergic and GABAergic neurotransmission, neuron morphology, and energy metabolism, ultimately affecting ADHD symptoms.

Comprehensive examination of changes in MC and associated molecular changes in the PFC enriches our understanding of the neurobiological basis of ADHD in the SHR animal model. These findings have implications for the development of targeted preclinical research aimed at discovering treatment methods that modulate immunological, redox, and metabolic pathways to alleviate ADHD symptoms and improve outcomes in patients.

## Data availability statement

The original contributions presented in the study are included in the article/Supplementary material, further inquiries can be directed to the corresponding author.

## Ethics statement

The animal study was approved by Local Ethics Committee for Animal Experimentation at the University of Warmia and Mazury in Olsztyn, Poland (permission number: no. 43/2014). Stringent adherence to the European Union Directive for animal experiments (2010/63/EU). The study was conducted in accordance with the local legislation and institutional requirements.

## Author contributions

EB-C: Conceptualization, Data curation, Formal analysis, Investigation, Methodology, Project administration, Validation, Visualization, Writing – original draft, Writing – review & editing. PW: Methodology, Writing – review & editing. MT: Funding acquisition, Supervision, Writing – review & editing. AH: Methodology, Writing – review & editing. AK: Conceptualization, Data curation, Funding acquisition, Investigation, Methodology, Project administration, Resources, Supervision, Validation, Visualization, Writing – review & editing.

## References

[ref1] Alaverdashvili M. Hackett M. J. Pickering I. J. Paterson P. G. (2014). Laminar-specific distribution of zinc: evidence for presence of layer IV in forelimb motor cortex in the rat. NeuroImage 103, 502–510. doi: 10.1016/j.neuroimage.2014.08.046, PMID: 25192655 PMC4490894

[ref2] Almeida L. G. Ricardo-Garcell J. Prado H. Barajas L. Fernández-Bouzas A. Avila D.. (2010). Reduced right frontal cortical thickness in children, adolescents and adults with ADHD and its correlation to clinical variables: a cross-sectional study. J. Psychiatr. Res. 44, 1214–1223. doi: 10.1016/j.jpsychires.2010.04.026, PMID: 20510424

[ref3] Archana E. Pai P. Prabhu B. K. Shenoy R. P. Prabhu K. Rao A. (2012). Altered biochemical parameters in saliva of pediatric attention deficit hyperactivity disorder. Neurochem. Res. 37, 330–334. doi: 10.1007/s11064-011-0616-x, PMID: 21964765

[ref4] Arkin M. R. Whitty A. (2009). The road less traveled: modulating signal transduction enzymes by inhibiting their protein-protein interactions. Curr. Opin. Chem. Biol. 13, 284–290. doi: 10.1016/j.cbpa.2009.05.125, PMID: 19553156

[ref5] Ashtari M. Kumra S. Bhaskar S. L. Clarke T. Thaden E. Cervellione K. L.. (2005). Attention-deficit/hyperactivity disorder: a preliminary diffusion tensor imaging study. Biol. Psychiatry 57, 448–455. doi: 10.1016/j.biopsych.2004.11.047, PMID: 15737658

[ref6] Baba S. P. Bhatnagar A. (2018). Role of THIOLS in oxidative stress. Curr. Opin. Toxicol. 7, 133–139. doi: 10.1016/j.cotox.2018.03.005, PMID: 30338308 PMC6188637

[ref7] Bandeira F. Lent R. Herculano-Houzel S. (2009). Changing numbers of neuronal and non-neuronal cells underlie postnatal brain growth in the rat. Proc. Natl. Acad. Sci. USA 106, 14108–14113. doi: 10.1073/pnas.0804650106, PMID: 19666520 PMC2729028

[ref8] Barthas F. Kwan A. C. (2017). Secondary motor cortex: where ‘sensory’ meets ‘motor’ in the rodent frontal cortex. Trends Neurosci. 40, 181–193. doi: 10.1016/j.tins.2016.11.006, PMID: 28012708 PMC5339050

[ref9] Batty M. J. Liddle E. B. Pitiot A. Toro R. Groom M. J. Scerif G.. (2010). Cortical gray matter in attention-deficit/hyperactivity disorder: a structural magnetic resonance imaging study. J. Am. Acad. Child Adolesc. Psychiatry 49, 229–238. doi: 10.1097/00004583-201003000-00006, PMID: 20410712 PMC2829134

[ref10] Bedwell S. A. Billett E. E. Crofts J. J. Tinsley C. J. (2023). The topology of connections between rat prefrontal, motor and sensory cortices. Front. Syst. Neurosci. 8:177. doi: 10.3389/fnsys.2014.00177 PMC4166227 25278850

[ref11] Bitsko R. H. Claussen A. H. Lichstein J. Black L. I. Jones S. E. Danielson M. L.. (2022). Mental health surveillance among children - United States, 2013-2019. MMWR Suppl. 71, 1–42. doi: 10.15585/mmwr.su7102a1, PMID: 35202359 PMC8890771

[ref12] Blazer L. L. Neubig R. R. (2009). Small molecule protein–protein interaction inhibitors as CNS therapeutic agents: current Progress and future hurdles. Neuropsychopharmacology 34, 126–141. doi: 10.1038/npp.2008.151, PMID: 18800065

[ref13] Blum K. Chen A. L. C. Braverman E. R. Comings D. E. Chen T. J. Arcuri V.. (2008). Attention-deficit-hyperactivity disorder and reward deficiency syndrome. Neuropsychiatr. Dis. Treat. 4, 893–918. doi: 10.2147/ndt.s2627, PMID: 19183781 PMC2626918

[ref14] Bollmann S. Ghisleni C. Poil S. S. Martin E. Ball J. Eich-Höchli D.. (2015). Developmental changes in gamma-aminobutyric acid levels in attention-deficit/hyperactivity disorder. Transl. Psychiatry 5:e589. doi: 10.1038/tp.2015.79, PMID: 26101852 PMC4490289

[ref15] Boriskin P. Gulenko O. Deviatkin A. Pavlova O. Toropovskiy A. (2019). Correlation of superoxide dismutase activity distribution in serum and tissues of small experimental animals. IOP Conf. Ser. Earth Environ. Sci. 403:012112. doi: 10.1088/1755-1315/403/1/012112

[ref16] Bostan A. C. Dum R. P. Strick P. L. (2013). Cerebellar networks with the cerebral cortex and basal ganglia. Trends Cogn. Sci. 17, 241–254. doi: 10.1016/j.tics.2013.03.003, PMID: 23579055 PMC3645327

[ref17] Bovaird J. H. Ngo T. T. Lenhoff H. M. (1982). Optimizing the o-phenylenediamine assay for horseradish peroxidase: effects of phosphate and pH, substrate and enzyme concentrations, and stopping reagents. Clin. Chem. 28, 2423–2426. doi: 10.1093/clinchem/28.12.2423, PMID: 6754137

[ref18] Bulut M. Selek S. Gergerlioglu H. S. Savas H. A. Yilmaz H. R. Yuce M.. (2007). Malondialdehyde levels in adult attention-deficit hyperactivity disorder. J. Psychiatry Neurosci. 32, 435–438, PMID: 18043768 PMC2077350

[ref19] Carissimi A. Martinez D. Kim L. J. Fiori C. Z. Vieira L. R. Rosa D. P.. (1999). Intermittent hypoxia, brain glyoxalase-1 and glutathione reductase-1, and anxiety-like behavior in mice. Rev. Bras. Psiquiatr. São Paulo Braz. 40, 376–381. doi: 10.1590/1516-4446-2017-2310 PMC6899376 30110090

[ref20] Carmona S. Vilarroya O. Bielsa A. Trèmols V. Soliva J. C. Rovira M.. (2005). Global and regional gray matter reductions in ADHD: a voxel-based morphometric study. Neurosci. Lett. 389, 88–93. doi: 10.1016/j.neulet.2005.07.020, PMID: 16129560

[ref21] Carraro E. Schilirò T. Biorci F. Romanazzi V. Degan R. Buonocore D.. (2018). Physical activity, lifestyle factors and oxidative stress in middle age healthy subjects. Int. J. Environ. Res. Public Health 15:1152. doi: 10.3390/ijerph15061152, PMID: 29865194 PMC6025138

[ref22] Castellanos F. X. Lee P. P. Sharp W. Jeffries N. O. Greenstein D. K. Clasen L. S.. (2002). Developmental trajectories of brain volume abnormalities in children and adolescents with attention-deficit/hyperactivity disorder. JAMA 288, 1740–1748. doi: 10.1001/jama.288.14.1740, PMID: 12365958

[ref23] Çelik V. K. Erşan E. Erşan S. Bakır S. Dogan O. (2013). Plasma catalase, glutathione-s-transferase and total antioxidant activity levels of children with attention deficit and hyperactivity disorder. Adv. Biosci. Biotechnol. 4, 183–187. doi: 10.4236/abb.2013.42026

[ref24] Ceylan M. Sener S. Bayraktar A. C. Kavutcu M. (2010). Oxidative imbalance in child and adolescent patients with attention-deficit/hyperactivity disorder. Prog. Neuro-Psychopharmacol. Biol. Psychiatry 34, 1491–1494. doi: 10.1016/j.pnpbp.2010.08.010, PMID: 20732373

[ref25] Ceylan M. F. Sener S. Bayraktar A. C. Kavutcu M. (2012). Changes in oxidative stress and cellular immunity serum markers in attention-deficit/hyperactivity disorder. Psychiatry Clin. Neurosci. 66, 220–226. doi: 10.1111/j.1440-1819.2012.02330.x, PMID: 22443244

[ref26] Chan KY Wasserman BP. (1993). Direct Colorimetric Assay of Free Thiol Groups and Disulfide Bonds in Suspensions of Solubilized and Particulate Cereal Proteins’

[ref27] Chen J. R. Hsu S. F. Hsu C. D. Hwang L. H. Yang S. C. (2004). Dietary patterns and blood fatty acid composition in children with attention-deficit hyperactivity disorder in Taiwan. J. Nutr. Biochem. 15, 467–472. doi: 10.1016/j.jnutbio.2004.01.008, PMID: 15302081

[ref28] Chen Y. Li Y. Hsieh T. Wang C. Cheng K. Wang L.. (2019). Aging-induced Akt activation involves in aging-related pathologies and Aβ-induced toxicity. Aging Cell 18:e12989. doi: 10.1111/acel.12989, PMID: 31183966 PMC6612704

[ref29] Chen X. Zhang Y. Wang H. Liu L. Li W. Xie P. (2022). The regulatory effects of lactic acid on neuropsychiatric disorders. Discov. Ment. Health 2:8. doi: 10.1007/s44192-022-00011-4, PMID: 37861858 PMC10501010

[ref30] Cheng R. Dhorajia V. V. Kim J. Kim Y. (2022). Mitochondrial iron metabolism and neurodegenerative diseases. Neurotoxicology 88, 88–101. doi: 10.1016/j.neuro.2021.11.003, PMID: 34748789 PMC8748425

[ref31] Chu E. Mychasiuk R. Hibbs M. L. Semple B. D. (2021). Dysregulated phosphoinositide 3-kinase signaling in microglia: shaping chronic neuroinflammation. J. Neuroinflammation 18:276. doi: 10.1186/s12974-021-02325-6 34838047 PMC8627624

[ref32] Coelho-Santos V. Cardoso F. L. Leitão R. A. Fontes-Ribeiro C. A. Silva A. P. (2018). Impact of developmental exposure to methylphenidate on rat brain’s immune privilege and behavior: control versus ADHD model. Brain Behav. Immun. 68, 169–182. doi: 10.1016/j.bbi.2017.10.016, PMID: 29061363

[ref33] Collings R. Harvey L. J. Hooper L. Hurst R. Brown T. J. Ansett J.. (2013). The absorption of iron from whole diets: a systematic review. Am. J. Clin. Nutr. 98, 65–81. doi: 10.3945/ajcn.112.050609, PMID: 23719560

[ref34] Corona J. C. (2020). Role of oxidative stress and Neuroinflammation in attention-deficit/hyperactivity disorder. Antioxidants 9:1039. doi: 10.3390/antiox9111039, PMID: 33114154 PMC7690797

[ref35] Coutinho A. E. Chapman K. E. (2011). The anti-inflammatory and immunosuppressive effects of glucocorticoids, recent developments and mechanistic insights. Mol. Cell. Endocrinol. 335, 2–13. doi: 10.1016/j.mce.2010.04.005, PMID: 20398732 PMC3047790

[ref36] Cowan K. Anichtchik O. Luo S. (2019). Mitochondrial integrity in neurodegeneration. CNS Neurosci. Ther. 25, 825–836. doi: 10.1111/cns.13105, PMID: 30746905 PMC6566061

[ref37] Derijk R. H. Schaaf M. J. Turner G. Datson N. A. Vreugdenhil E. Cidlowski J.. (2001). A human glucocorticoid receptor gene variant that increases the stability of the glucocorticoid receptor beta-isoform mRNA is associated with rheumatoid arthritis. J. Rheumatol. 28, 2383–2388, PMID: 11708406

[ref38] DeVito J. L. Graham J. Sackett G. P. (1989). Volumetric growth of the major brain divisions in fetal *Macaca nemestrina*. J. Hirnforsch. 30, 479–487, PMID: 2794488

[ref39] Donfrancesco R. Nativio P. Di Benedetto A. Villa M. P. Andriola E. Melegari M. G.. (2020). Anti-Yo antibodies in children with ADHD: first results about serum cytokines. J. Atten. Disord. 24, 1497–1502. doi: 10.1177/1087054716643387, PMID: 27095560

[ref40] Dunn G. A. Nigg J. T. Sullivan E. L. (2019). Neuroinflammation as a risk factor for attention deficit hyperactivity disorder. Pharmacol. Biochem. Behav. 182, 22–34. doi: 10.1016/j.pbb.2019.05.005, PMID: 31103523 PMC6855401

[ref41] Dutra T. G. Baltar A. Monte-Silva K. K. (2016). Motor cortex excitability in attention-deficit hyperactivity disorder (ADHD): a systematic review and meta-analysis. Res. Dev. Disabil. 56, 1–9. doi: 10.1016/j.ridd.2016.01.022, PMID: 27240241

[ref42] Eislmayr K. Bestehorn A. Morelli L. Borroni M. Walle L. V. Lamkanfi M.. Nonredundancy of IL-1α and IL-1β is defined by distinct regulation of tissues orchestrating resistance versus tolerance to infection. Sci. Adv. 8:eabj7293. doi: 10.1126/sciadv.abj7293 PMC8890706 35235356

[ref43] Elia J. Ambrosini P. Berrettini W. (2008). ADHD characteristics: I. Concurrent co-morbidity patterns in children & adolescents. Child Adolesc. Psychiatry Ment. Health 2:15. doi: 10.1186/1753-2000-2-15 18598351 PMC2500004

[ref44] Erejuwa O. O. Sulaiman S. A. Ab Wahab M. S. Sirajudeen K. N. S. Salleh S. Gurtu S. (2012). Honey supplementation in spontaneously hypertensive rats elicits antihypertensive effect via amelioration of renal oxidative stress. Oxidative Med. Cell. Longev. 2012:374037, 1–14. doi: 10.1155/2012/374037 PMC3270456 22315654

[ref45] Erickson K. Drevets W. Schulkin J. (2003). Glucocorticoid regulation of diverse cognitive functions in normal and pathological emotional states. Neurosci. Biobehav. Rev. 27, 233–246. doi: 10.1016/S0149-7634(03)00033-2, PMID: 12788335

[ref46] Ernst M. Liebenauer L. L. King A. C. Fitzgerald G. A. Cohen R. M. Zametkin A. J. (1994). Reduced brain metabolism in hyperactive girls. J. Am. Acad. Child Adolesc. Psychiatry 33, 858–868. doi: 10.1097/00004583-199407000-00012 8083143

[ref47] Eskandari R. Abdullah O. Mason C. Lloyd K. E. Oeschle A. N. McAllister J. P. (2014). Differential vulnerability of white matter structures to experimental infantile hydrocephalus detected by diffusion tensor imaging. Childs Nerv. Syst. 30, 1651–1661. doi: 10.1007/s00381-014-2500-x, PMID: 25070594

[ref48] Ferreira A. Neves P. Gozzelino R. (2019). Multilevel impacts of Iron in the brain: the cross talk between neurophysiological mechanisms, cognition, and social behavior. Pharmaceuticals 12:126. doi: 10.3390/ph12030126, PMID: 31470556 PMC6789770

[ref49] Forero D. A. Arboleda G. H. Vasquez R. Arboleda H. (2009). Candidate genes involved in neural plasticity and the risk for attention-deficit hyperactivity disorder: a meta-analysis of 8 common variants. J. Psychiatry Neurosci. 34, 361–366, PMID: 19721846 PMC2732742

[ref50] Fremont R. Dworkin J. Manoochehri M. Krueger F. Huey E. Grafman J. (2022). Damage to the dorsolateral prefrontal cortex is associated with repetitive compulsive behaviors in patients with penetrating brain injury. BMJ Neurol. Open 4:e000229. doi: 10.1136/bmjno-2021-000229, PMID: 35519903 PMC9020295

[ref51] Fuster J. M. (2002). Frontal lobe and cognitive development. J. Neurocytol. 31, 373–385. doi: 10.1023/A:1024190429920 12815254

[ref52] Gao Y. Shuai D. Bu X. Hu X. Tang S. Zhang L.. (2019). Impairments of large-scale functional networks in attention-deficit/hyperactivity disorder: a meta-analysis of resting-state functional connectivity. Psychol. Med. 49, 2475–2485. doi: 10.1017/S003329171900237X, PMID: 31500674

[ref53] Gao J. Wang T. Wang C. Wang S. Wang W. Ma D.. (2019). Effects of Tianshu capsule on spontaneously hypertensive rats as revealed by 1H-NMR-based metabolic profiling. Front. Pharmacol. 10:989. doi: 10.3389/fphar.2019.00989 31572179 PMC6749043

[ref54] Garcia-Rizo C. Kirkpatrick B. Fernandez-Egea E. Oliveira C. Bernardo M. (2016). Abnormal glycemic homeostasis at the onset of serious mental illnesses: a common pathway. Psychoneuroendocrinology 67, 70–75. doi: 10.1016/j.psyneuen.2016.02.001, PMID: 26878465 PMC4844848

[ref55] García-Yébenes I. Sobrado M. Moraga A. Zarruk J. G. Romera V. G. Pradillo J. M.. (2012). Iron overload, measured as serum ferritin, increases brain damage induced by focal ischemia and early reperfusion. Neurochem. Int. 61, 1364–1369. doi: 10.1016/j.neuint.2012.09.014, PMID: 23036361

[ref56] Gaub M. Carlson C. L. (1997). Gender differences in ADHD: a meta-analysis and critical review. J. Am. Acad. Child Adolesc. Psychiatry 36, 1036–1045. doi: 10.1097/00004583-199708000-00011, PMID: 9256583

[ref57] Gilbert D. L. Huddleston D. A. Wu S. W. Pedapati E. V. Horn P. S. Hirabayashi K.. (2019). Motor cortex inhibition and modulation in children with ADHD. Neurology 93, e599–e610. doi: 10.1212/WNL.0000000000007899, PMID: 31315973 PMC6709998

[ref58] Grafton S. T. Volz L. J. (2019). From ideas to action: the prefrontal-premotor connections that shape motor behavior. Handb. Clin. Neurol. 163, 237–255. doi: 10.1016/B978-0-12-804281-6.00013-6, PMID: 31590733

[ref59] Griffiths K. R. Grieve S. M. Kohn M. R. Clarke S. Williams L. M. Korgaonkar M. S. (2016). Altered gray matter organization in children and adolescents with ADHD: a structural covariance connectome study. Transl. Psychiatry 6:e947. doi: 10.1038/tp.2016.219, PMID: 27824356 PMC5314130

[ref60] Guo C. Sun L. Chen X. Zhang D. (2013). Oxidative stress, mitochondrial damage and neurodegenerative diseases. Neural Regen. Res. 8, 2003–2014. doi: 10.3969/j.issn.1673-5374.2013.21.009, PMID: 25206509 PMC4145906

[ref61] Ha S. Lee H. Choi Y. Kang H. Jeon S. J. Ryu J. H.. (2020). Maturational delay and asymmetric information flow of brain connectivity in SHR model of ADHD revealed by topological analysis of metabolic networks. Sci. Rep. 10:3197. doi: 10.1038/s41598-020-59921-4, PMID: 32081992 PMC7035354

[ref62] Habig W. H. Pabst M. J. Jakoby W. B. (1974). Glutathione S-transferases: the first enzymatic step in mercapturic acid formation. J. Biol. Chem. 249, 7130–7139. doi: 10.1016/S0021-9258(19)42083-8 4436300

[ref63] Han D. Williams E. Cadenas E. (2001). Mitochondrial respiratory chain-dependent generation of superoxide anion and its release into the intermembrane space. Biochem. J. 353, 411–416. doi: 10.1042/bj3530411, PMID: 11139407 PMC1221585

[ref64] Hoogman M. Muetzel R. Guimaraes J. P. Shumskaya E. Mennes M. Zwiers M. P.. (2019). Brain imaging of the cortex in ADHD: a coordinated analysis of large-scale clinical and population-based samples. Am. J. Psychiatry 176, 531–542. doi: 10.1176/appi.ajp.2019.18091033, PMID: 31014101 PMC6879185

[ref65] Horowitz M. P. Greenamyre J. T. (2010). Mitochondrial Iron metabolism and its role in neurodegeneration. J. Alzheimers Dis. 20, S551–S568. doi: 10.3233/JAD-2010-100354, PMID: 20463401 PMC3085540

[ref66] Hsieh Y. L. Yang C. C. (2008). Age-series characteristics of locomotor activities in spontaneously hypertensive rats: a comparison with the Wistar-Kyoto strain. Physiol. Behav. 93, 777–782. doi: 10.1016/j.physbeh.2007.11.032, PMID: 18155738

[ref67] Hsu J. W. Lee L. C. Chen R. F. Yen C. T. Chen Y. S. Tsai M. L. (2010). Striatal volume changes in a rat model of childhood attention-deficit/hyperactivity disorder. Psychiatry Res. 179, 338–341. doi: 10.1016/j.psychres.2009.08.008 20493538

[ref68] Imai H. Yamamoto T. Katsuyama Y. Kikkawa S. Terashima T. (2012). Subcortically and callosally projecting neurons are distinct neuronal pools in the motor cortex of the reeler mouse. Kobe J. Med. Sci. 58, E86–E95, PMID: 23143474

[ref69] Jeong M. Kim Y. Kim J. Ferrante D. D. Mitra P. P. Osten P.. (2016). Comparative three-dimensional connectome map of motor cortical projections in the mouse brain. Sci. Rep. 6:20072. doi: 10.1038/srep20072, PMID: 26830143 PMC4735720

[ref70] Ježek P. Jabůrek M. Holendová B. Engstová H. Dlasková A. (2023). Mitochondrial cristae morphology reflecting metabolism, superoxide formation, redox homeostasis, and pathology. Antioxid. Redox Signal. 39, 635–683. doi: 10.1089/ars.2022.0173, PMID: 36793196 PMC10615093

[ref71] Jing B. Liu B. Li H. Lei J. Wang Z. Yang Y.. (2018). Within-subject test-retest reliability of the atlas-based cortical volume measurement in the rat brain: a voxel-based morphometry study. J. Neurosci. Methods 307, 46–52. doi: 10.1016/j.jneumeth.2018.06.022, PMID: 29960027 PMC6461491

[ref72] Johansen E. B. Killeen P. R. Sagvolden T. (2007). Behavioral variability, elimination of responses, and delay-of-reinforcement gradients in SHR and WKY rats. Behav. Brain Funct. 3:60. doi: 10.1186/1744-9081-3-60, PMID: 18028539 PMC2219961

[ref73] Johansen E. B. Sagvolden T. Kvande G. (2005). Effects of delayed reinforcers on the behavior of an animal model of attention-deficit/hyperactivity disorder (ADHD). Behav. Brain Res. 162, 47–61. doi: 10.1016/j.bbr.2005.02.034, PMID: 15922066

[ref74] Jugé L. Pong A. C. Bongers A. Sinkus R. Bilston L. E. Cheng S. (2016). Changes in rat brain tissue microstructure and stiffness during the development of experimental obstructive hydrocephalus. PLoS One 11:e0148652. doi: 10.1371/journal.pone.0148652, PMID: 26848844 PMC4743852

[ref75] Juneja M. Jain R. Singh V. Mallika V. (2010). Iron deficiency in Indian children with attention deficit hyperactivity disorder. Indian Pediatr. 47, 955–958. doi: 10.1007/s13312-010-0160-9, PMID: 20453262

[ref76] Karababa İ. F. Savas S. N. Selek S. Cicek E. Cicek E. I. Asoglu M.. (2017). Homocysteine levels and oxidative stress parameters in patients with adult ADHD. J. Atten. Disord. 21, 487–493. doi: 10.1177/1087054714538657, PMID: 24994877

[ref77] Karalis V. Bateup H. S. (2021). Current approaches and future directions for the treatment of mTORopathies. Dev. Neurosci. 43, 143–158. doi: 10.1159/000515672, PMID: 33910214 PMC8440338

[ref78] Kates W. R. Frederikse M. Mostofsky S. H. Folley B. S. Cooper K. Mazur-Hopkins P.. (2002). MRI parcellation of the frontal lobe in boys with attention deficit hyperactivity disorder or Tourette syndrome. Psychiatry Res. 116, 63–81. doi: 10.1016/S0925-4927(02)00066-5 12426035

[ref79] Kim D. Yadav D. Song M. (2024). An updated review on animal models to study attention-deficit hyperactivity disorder. Transl. Psychiatry 14, 1–12. doi: 10.1038/s41398-024-02893-0 38605002 PMC11009407

[ref80] Knight T. R. Choudhuri S. Klaassen C. D. (2007). Constitutive mRNA expression of various glutathione S-transferase isoforms in different tissues of mice. Toxicol. Sci. Off. J. Soc. Toxicol. 100, 513–524. doi: 10.1093/toxsci/kfm233, PMID: 17890767

[ref81] Koh G. C. K. W. Porras P. Aranda B. Hermjakob H. Orchard S. E. (2012). Analyzing protein-protein interaction networks. J. Proteome Res. 11, 2014–2031. doi: 10.1021/pr201211w 22385417

[ref82] Kolb B. Gibb R. (2011). Brain plasticity and behaviour in the developing brain. J. Can. Acad. Child Adolesc. Psychiatry 20:265. 22114608 PMC3222570

[ref83] Kozłowska A. Wojtacha P. Majewski M. Równiak M. (2019a). The cytokine alterations/abnormalities and oxidative damage in the pancreas during hypertension development. Pflugers Arch. 471, 1331–1340. doi: 10.1007/s00424-019-02312-0, PMID: 31624954 PMC6814849

[ref84] Kozłowska A. Wojtacha P. Równiak M. Kolenkiewicz M. Huang A. C. W. (2019b). ADHD pathogenesis in the immune, endocrine and nervous systems of juvenile and maturating SHR and WKY rats. Psychopharmacology 236, 2937–2958. doi: 10.1007/s00213-019-5180-0, PMID: 30737597 PMC6820808

[ref85] Kozłowska A. Wojtacha P. Równiak M. Kolenkiewicz M. Tsai M. L. (2019c). Differences in serum steroid hormones concentrations in spontaneously hypertensive rats (SHR) - an animal model of attention-deficit/hyperactivity disorder (ADHD). Physiol. Res. 68, 25–36. doi: 10.33549/physiolres.933907, PMID: 30433797

[ref86] Kriegstein A. R. Dichter M. A. (1983). Morphological classification of rat cortical neurons in cell culture. J. Neurosci. 3, 1634–1647. doi: 10.1523/JNEUROSCI.03-08-01634.1983, PMID: 6875660 PMC6564534

[ref87] Laube C. van den Bos W. Fandakova Y. (2020). The relationship between pubertal hormones and brain plasticity: implications for cognitive training in adolescence. Dev. Cogn. Neurosci. 42:100753. doi: 10.1016/j.dcn.2020.100753, PMID: 32072931 PMC7005587

[ref88] Lee D. Y. (2015). Roles of mTOR signaling in brain development. Exp. Neurobiol. 24, 177–185. doi: 10.5607/en.2015.24.3.177, PMID: 26412966 PMC4580744

[ref89] Lee K. H. Cha M. Lee B. H. (2020). Neuroprotective effect of antioxidants in the brain. Int. J. Mol. Sci. 21:7152. doi: 10.3390/ijms21197152, PMID: 32998277 PMC7582347

[ref90] Lee C. Kim Y. Kaang B. K. (2022). The primary motor cortex: the hub of motor learning in rodents. Neuroscience 485, 163–170. doi: 10.1016/j.neuroscience.2022.01.009, PMID: 35051529

[ref91] Leffa D. T. Bellaver B. de Oliveira C. de Macedo I. C. de Freitas J. S. Grevet E. H.. (2017). Increased oxidative parameters and decreased cytokine levels in an animal model of attention-deficit/hyperactivity disorder. Neurochem. Res. 42, 3084–3092. doi: 10.1007/s11064-017-2341-6, PMID: 28664398

[ref92] Leffa D. T. Panzenhagen A. C. Salvi A. A. Bau C. H. D. Pires G. N. Torres I. L. S.. (2019). Systematic review and meta-analysis of the behavioral effects of methylphenidate in the spontaneously hypertensive rat model of attention-deficit/hyperactivity disorder. Neurosci. Biobehav. Rev. 100, 166–179. doi: 10.1016/j.neubiorev.2019.02.019, PMID: 30826386

[ref93] Leffa D. T. Torres I. L. S. Rohde L. A. (2018). A review on the role of inflammation in attention-deficit/hyperactivity disorder. Neuroimmunomodulation 25, 328–333. doi: 10.1159/000489635 29874674

[ref94] Li D. Li T. Niu Y. Xiang J. Cao R. Liu B.. (2019). Reduced hemispheric asymmetry of brain anatomical networks in attention deficit hyperactivity disorder. Brain Imaging Behav. 13, 669–684. doi: 10.1007/s11682-018-9881-5, PMID: 29752654

[ref95] Liu J. Fan W. Jia Y. Su X. Wu W. Long X.. (2020). Altered gray matter volume in patients with type 1 diabetes mellitus. Front. Endocrinol. 11:45. doi: 10.3389/fendo.2020.00045, PMID: 32117070 PMC7031205

[ref96] Liu J. Liu D. Pu X. Zou K. Xie T. Li Y.. (2023). The secondary motor cortex-striatum circuit contributes to suppressing inappropriate responses in perceptual decision behavior. Neurosci. Bull. 39, 1544–1560. doi: 10.1007/s12264-023-01073-2, PMID: 37253985 PMC10533474

[ref97] Liu Q. Qiu J. Liang M. Golinski J. van Leyen K. Jung J. E.. (2014). Akt and mTOR mediate programmed necrosis in neurons. Cell Death Dis. 5:e1084. doi: 10.1038/cddis.2014.69, PMID: 24577082 PMC3944276

[ref98] Lolansen S. D. Barbuskaite D. Ye F. Xiang J. Keep R. F. MacAulay N. (2023). Spontaneously hypertensive rats can become hydrocephalic despite undisturbed secretion and drainage of cerebrospinal fluid. Fluids Barriers CNS 20:53. doi: 10.1186/s12987-023-00448-x, PMID: 37403103 PMC10318838

[ref99] Loregian A. Palù G. (2005). Disruption of protein-protein interactions: towards new targets for chemotherapy. J. Cell. Physiol. 204, 750–762. doi: 10.1002/jcp.20356, PMID: 15880642

[ref100] Lu H. Zhou Q. He J. Jiang Z. Peng C. Tong R.. (2020). Recent advances in the development of protein–protein interactions modulators: mechanisms and clinical trials. Signal Transduct. Target. Ther. 5:213. doi: 10.1038/s41392-020-00315-3, PMID: 32968059 PMC7511340

[ref101] Magdalon J. Sánchez-Sánchez S. M. Griesi-Oliveira K. Sertié A. L. (2017). Dysfunctional mTORC1 signaling: a convergent mechanism between syndromic and nonsyndromic forms of autism Spectrum disorder? Int. J. Mol. Sci. 18:659. doi: 10.3390/ijms18030659, PMID: 28335463 PMC5372671

[ref102] Magistretti P. J. Allaman I. (2018). Lactate in the brain: from metabolic end-product to signalling molecule. Nat. Rev. Neurosci. 19, 235–249. doi: 10.1038/nrn.2018.19, PMID: 29515192

[ref103] Manor I. Magen A. Keidar D. Rosen S. Tasker H. Cohen T.. (2012). The effect of phosphatidylserine containing Omega3 fatty-acids on attention-deficit hyperactivity disorder symptoms in children: a double-blind placebo-controlled trial, followed by an open-label extension. Eur. Psychiatry J. Assoc. Eur. Psychiatr. 27, 335–342. doi: 10.1016/j.eurpsy.2011.05.004, PMID: 21807480

[ref104] McBean G. J. López M. G. Wallner F. K. (2017). Redox-based therapeutics in neurodegenerative disease. Br. J. Pharmacol. 174, 1750–1770. doi: 10.1111/bph.13551, PMID: 27477685 PMC5446580

[ref105] McGill M. R. (2016). The past and present of serum aminotransferases and the future of liver injury biomarkers. Excli J. 15, 817–828. doi: 10.17179/excli2016-800, PMID: 28337112 PMC5318690

[ref106] Medin T. Medin H. Hefte M. B. Storm-Mathisen J. Bergersen L. H. (2019). Upregulation of the lactate transporter monocarboxylate transporter 1 at the blood-brain barrier in a rat model of attention-deficit/hyperactivity disorder suggests hyperactivity could be a form of self-treatment. Behav. Brain Res. 360, 279–285. doi: 10.1016/j.bbr.2018.12.023, PMID: 30550949

[ref107] Meiser J. Weindl D. Hiller K. (2013). Complexity of dopamine metabolism. Cell Commun. Signal 11:34. doi: 10.1186/1478-811X-11-34, PMID: 23683503 PMC3693914

[ref108] Miller A. H. Haroon E. Raison C. L. Felger J. C. (2013). Cytokine targets in the brain: impact on neurotransmitters and neurocircuits. Depress. Anxiety 30, 297–306. doi: 10.1002/da.22084, PMID: 23468190 PMC4141874

[ref109] Misiak B. Łoniewski I. Marlicz W. Frydecka D. Szulc A. Rudzki L.. (2020). The HPA axis dysregulation in severe mental illness: can we shift the blame to gut microbiota? Prog. Neuro Psychopharmacol. Biol. Psychiatry 102:109951. doi: 10.1016/j.pnpbp.2020.109951, PMID: 32335265

[ref110] Mostofsky S. H. Cooper K. L. Kates W. R. Denckla M. B. Kaufmann W. E. (2002). Smaller prefrontal and premotor volumes in boys with attention-deficit/hyperactivity disorder. Biol. Psychiatry 52, 785–794. doi: 10.1016/S0006-3223(02)01412-9, PMID: 12372650

[ref111] Mostofsky S. H. Rimrodt S. L. Schafer J. G. B. Boyce A. Goldberg M. C. Pekar J. J.. (2006). Atypical motor and sensory cortex activation in attention-deficit/hyperactivity disorder: a functional magnetic resonance imaging study of simple sequential finger tapping. Biol. Psychiatry 59, 48–56. doi: 10.1016/j.biopsych.2005.06.011, PMID: 16139806

[ref112] Mullen R. J. Buck C. R. Smith A. M. (1992). NeuN, a neuronal specific nuclear protein in vertebrates. Dev Camb Engl. 116, 201–211. doi: 10.1242/dev.116.1.201 1483388

[ref113] Muñoz-Castañeda R. Zingg B. Matho K. S. Chen X. Wang Q. Foster N. N.. (2021). Cellular anatomy of the mouse primary motor cortex. Nature 598, 159–166. doi: 10.1038/s41586-021-03970-w, PMID: 34616071 PMC8494646

[ref114] Navas F. J. Córdova A. (2000). Iron distribution in different tissues in rats following exercise. Biol. Trace Elem. Res. 73, 259–268. doi: 10.1385/BTER:73:3:259, PMID: 11049216

[ref115] Naya N. Tsuji T. Nishigaki N. Sakai C. Chen Y. Jung S.. (2021). The burden of undiagnosed adults with attention-deficit/hyperactivity disorder symptoms in Japan: a cross-sectional study. Cureus 13:e19615. doi: 10.7759/cureus.19615, PMID: 34956750 PMC8674614

[ref116] Nero T. L. Morton C. J. Holien J. K. Wielens J. Parker M. W. (2014). Oncogenic protein interfaces: small molecules, big challenges. Nat. Rev. Cancer 14, 248–262. doi: 10.1038/nrc3690, PMID: 24622521

[ref117] Oades R. D. Myint A. M. Dauvermann M. R. Schimmelmann B. G. Schwarz M. J. (2010). Attention-deficit hyperactivity disorder (ADHD) and glial integrity: an exploration of associations of cytokines and kynurenine metabolites with symptoms and attention. Behav. Brain Funct. 6:32. doi: 10.1186/1744-9081-6-32, PMID: 20534153 PMC2900218

[ref118] Öğütlü H. Mertoğlu C. Gök G. Neşelioğlu S. (2020). Thiols and ceruloplasmin levels in serum of children with attention deficit hyperactivity disorder: a cross-sectional study. Psychiatry Res. 294:113546. doi: 10.1016/j.psychres.2020.113546, PMID: 33160216

[ref119] Oztop D. Altun H. Baskol G. Ozsoy S. (2012). Oxidative stress in children with attention deficit hyperactivity disorder. Clin. Biochem. 45, 745–748. doi: 10.1016/j.clinbiochem.2012.03.027 22497926

[ref120] Pace T. W. W. Miller A. H. (2009). Cytokines and glucocorticoid receptor signaling. Relevance to major depression. Ann. N. Y. Acad. Sci. 1179, 86–105. doi: 10.1111/j.1749-6632.2009.04984.x, PMID: 19906234 PMC3399249

[ref121] Paloyelis Y. Mehta M. A. Kuntsi J. Asherson P. (2007). Functional magnetic resonance imaging in attention deficit hyperactivity disorder (ADHD): a systematic literature review. Expert. Rev. Neurother. 7, 1337–1356. doi: 10.1586/14737175.7.10.1337, PMID: 17939771 PMC3763932

[ref122] Park S. Shin J. Hong Y. Kim S. Lee S. Park K.. (2012). Forced exercise enhances functional recovery after focal cerebral ischemia in spontaneously hypertensive rats. Brain Sci. 2, 483–503. doi: 10.3390/brainsci2040483, PMID: 24961257 PMC4061815

[ref123] Paxinos G. Watson C. (2005). The rat brain in stereotaxic coordinates [internet]. 5th Edn. Amsterdam: Elsevier Academic Press.

[ref124] Perera H. Jeewandara K. C. Seneviratne S. Guruge C. (2012). Combined ω3 and ω6 supplementation in children with attention-deficit hyperactivity disorder (ADHD) refractory to methylphenidate treatment: a double-blind, placebo-controlled study. J. Child Neurol. 27, 747–753. doi: 10.1177/0883073811435243 22596014

[ref126] Pliszka S. R. (2005). The neuropsychopharmacology of attention-deficit/hyperactivity disorder. Biol. Psychiatry 57, 1385–1390. doi: 10.1016/j.biopsych.2004.08.026 15950012

[ref127] Porcher L. Bruckmeier S. Burbano S. D. Finnell J. E. Gorny N. Klett J.. (2021). Aging triggers an upregulation of a multitude of cytokines in the male and especially the female rodent hippocampus but more discrete changes in other brain regions. J. Neuroinflammation 18:219. doi: 10.1186/s12974-021-02252-6, PMID: 34551810 PMC8459490

[ref129] Proal E. Reiss P. T. Klein R. G. Mannuzza S. Gotimer K. Ramos-Olazagasti M. A.. (2011). Brain gray matter deficits at 33-year follow-up in adults with attention-deficit/hyperactivity disorder established in childhood. Arch. Gen. Psychiatry 68, 1122–1134. doi: 10.1001/archgenpsychiatry.2011.117, PMID: 22065528 PMC3554238

[ref130] Puurunen J. Sulkama S. Tiira K. Araujo C. Lehtonen M. Hanhineva K.. (2016). A non-targeted metabolite profiling pilot study suggests that tryptophan and lipid metabolisms are linked with ADHD-like behaviours in dogs. Behav. Brain Funct. 12:27. doi: 10.1186/s12993-016-0112-1, PMID: 27686065 PMC5043524

[ref131] Quinn R. (2005). Comparing rat’s to human’s age: how old is my rat in people years? Nutr Burbank Los Angel Cty Calif. 21, 775–777. doi: 10.1016/j.nut.2005.04.002 15925305

[ref132] Ramtekkar U. P. Reiersen A. M. Todorov A. A. Todd R. D. (2010). Sex and age differences in attention-deficit/hyperactivity disorder symptoms and diagnoses: implications for DSM-V and ICD-11. J. Am. Acad. Child Adolesc. Psychiatry 49, 217–228.e3. doi: 10.1016/j.jaac.2009.11.011, PMID: 20410711 PMC3101894

[ref133] Richardson A. J. Puri B. K. (2002). A randomized double-blind, placebo-controlled study of the effects of supplementation with highly unsaturated fatty acids on ADHD-related symptoms in children with specific learning difficulties. Prog. Neuro Psychopharmacol. Biol. Psychiatry 26, 233–239. doi: 10.1016/S0278-5846(01)00254-8, PMID: 11817499

[ref134] Rossignol D. A. Frye R. E. (2012). Mitochondrial dysfunction in autism spectrum disorders: a systematic review and meta-analysis. Mol. Psychiatry 17, 290–314. doi: 10.1038/mp.2010.136, PMID: 21263444 PMC3285768

[ref135] Sagvolden T. Johansen E. B. (2012). Rat models of ADHD. Curr. Top. Behav. Neurosci. 9, 301–315. doi: 10.1007/7854_2011_126 21487952

[ref136] Sagvolden T. Johansen E. B. Wøien G. Walaas S. I. Storm-Mathisen J. Bergersen L. H.. (2009). The spontaneously hypertensive rat model of ADHD – the importance of selecting the appropriate reference strain. Neuropharmacology 57, 619–626. doi: 10.1016/j.neuropharm.2009.08.004, PMID: 19698722 PMC2783904

[ref137] Sagvolden T. Russell V. A. Aase H. Johansen E. B. Farshbaf M. (2005). Rodent models of attention-deficit/hyperactivity disorder. Biol. Psychiatry 57, 1239–1247. doi: 10.1016/j.biopsych.2005.02.002 15949994

[ref138] Salari N. Ghasemi H. Abdoli N. Rahmani A. Shiri M. H. Hashemian A. H.. (2023). The global prevalence of ADHD in children and adolescents: a systematic review and meta-analysis. Ital. J. Pediatr. 49:48. doi: 10.1186/s13052-023-01456-1, PMID: 37081447 PMC10120242

[ref139] Sánchez-Alegría K. Flores-León M. Avila-Muñoz E. Rodríguez-Corona N. Arias C. (2018). PI3K signaling in neurons: a central node for the control of multiple functions. Int. J. Mol. Sci. 19:3725. doi: 10.3390/ijms19123725, PMID: 30477115 PMC6321294

[ref140] Schellekens W. Bakker C. Ramsey N. F. Petridou N. (2022). Moving in on human motor cortex. Characterizing the relationship between body parts with non-rigid population response fields. PLoS Comput. Biol. 18:e1009955. doi: 10.1371/journal.pcbi.1009955, PMID: 35377877 PMC9009778

[ref141] Schindelin J. Arganda-Carreras I. Frise E. Kaynig V. Longair M. Pietzsch T.. (2012). Fiji - an open source platform for biological image analysis. Nat. Methods 9, 676–682. doi: 10.1038/nmeth.2019 22743772 PMC3855844

[ref142] Schmitz F. Scherer E. B. S. Machado F. R. da Cunha A. A. Tagliari B. Netto C. A.. (2012). Methylphenidate induces lipid and protein damage in prefrontal cortex, but not in cerebellum, striatum and hippocampus of juvenile rats. Metab. Brain Dis. 27, 605–612. doi: 10.1007/s11011-012-9335-5 22968482

[ref143] Seitz C. Hugle M. Cristofanon S. Tchoghandjian A. Fulda S. (2013). The dual PI3K/mTOR inhibitor NVP-BEZ235 and chloroquine synergize to trigger apoptosis via mitochondrial-lysosomal cross-talk. Int. J. Cancer 132, 2682–2693. doi: 10.1002/ijc.27935, PMID: 23151917

[ref144] Selek S. Bulut M. Ocak A. R. Kalenderoğlu A. Savaş H. A. (2012). Evaluation of total oxidative status in adult attention deficit hyperactivity disorder and its diagnostic implications. J. Psychiatr. Res. 46, 451–455. doi: 10.1016/j.jpsychires.2011.12.007, PMID: 22257388

[ref145] Semple B. D. Blomgren K. Gimlin K. Ferriero D. M. Noble-Haeusslein L. J. (2013). Brain development in rodents and humans: identifying benchmarks of maturation and vulnerability to injury across species. Prog. Neurobiol. 106-107, 1–16. doi: 10.1016/j.pneurobio.2013.04.001 23583307 PMC3737272

[ref146] Shaw P. Eckstrand K. Sharp W. Blumenthal J. Lerch J. P. Greenstein D.. (2007). Attention-deficit/hyperactivity disorder is characterized by a delay in cortical maturation. Proc. Natl. Acad. Sci. USA 104, 19649–19654. doi: 10.1073/pnas.0707741104, PMID: 18024590 PMC2148343

[ref147] Shaw P. Malek M. Watson B. Sharp W. Evans A. Greenstein D. (2012). Development of cortical surface area and gyrification in attention-deficit/hyperactivity disorder. Biol. Psychiatry 72, 191–197. doi: 10.1016/j.biopsych.2012.01.031, PMID: 22418014 PMC10376909

[ref148] Sibley M. H. Mitchell J. T. Becker S. P. (2016). Method of adult diagnosis influences estimated persistence of childhood ADHD: a systematic review of longitudinal studies. Lancet Psychiatry 3, 1157–1165. doi: 10.1016/S2215-0366(16)30190-0, PMID: 27745869

[ref149] Sibley M. H. Pelham W. E. Molina B. S. G. Gnagy E. M. Waxmonsky J. G. Waschbusch D. A.. (2012). When diagnosing ADHD in young adults emphasize informant reports, DSM items, and impairment. J. Consult. Clin. Psychol. 80, 1052–1061. doi: 10.1037/a0029098, PMID: 22774792 PMC3919146

[ref150] Skalny A. V. Mazaletskaya A. L. Zaitseva I. P. Skalny A. A. Spandidos D. A. Tsatsakis A.. (2021). Alterations in serum amino acid profiles in children with attention deficit/hyperactivity disorder. Biomed. Rep. 14:47. doi: 10.3892/br.2021.1423, PMID: 33786176 PMC7995246

[ref151] Sobowale O. A. Parry-Jones A. R. Smith C. J. Tyrrell P. J. Rothwell N. J. Allan S. M. (2016). Interleukin-1 in stroke: from bench to bedside. Stroke 47, 2160–2167. doi: 10.1161/STROKEAHA.115.010001, PMID: 26931154

[ref152] Soma S. Saiki A. Yoshida J. Ríos A. Kawabata M. Sakai Y.. (2017). Distinct laterality in forelimb-movement representations of rat primary and secondary motor cortical neurons with Intratelencephalic and pyramidal tract projections. J. Neurosci. 37, 10904–10916. doi: 10.1523/JNEUROSCI.1188-17.2017, PMID: 28972128 PMC6596487

[ref153] Song Y. Yuan H. Chen T. Lu M. Lei S. Han X. (2021). An Shen Ding Zhi Ling alleviates symptoms of attention deficit hyperactivity disorder via anti-inflammatory effects in spontaneous hypertensive rats. Front. Pharmacol. 11:617581. doi: 10.3389/fphar.2020.617581, PMID: 33536923 PMC7847841

[ref154] Spear L. P. (2000). The adolescent brain and age-related behavioral manifestations. Neurosci. Biobehav. Rev. 24, 417–463. doi: 10.1016/S0149-7634(00)00014-2 10817843

[ref155] Stefano G. B. Challenger S. Kream R. M. (2016). Hyperglycemia-associated alterations in cellular signaling and dysregulated mitochondrial bioenergetics in human metabolic disorders. Eur. J. Nutr. 55, 2339–2345. doi: 10.1007/s00394-016-1212-2, PMID: 27084094 PMC5122622

[ref156] Sutcubasi Kaya B. Metin B. Tas Z. C. Buyukaslan A. Soysal A. Hatiloglu D.. (2018). Gray matter increase in motor cortex in pediatric ADHD: a voxel-based morphometry study. J. Atten. Disord. 22, 611–618. doi: 10.1177/1087054716659139, PMID: 27469397

[ref157] Szczepanski S. M. Knight R. T. (2014). Insights into human behavior from lesions to the prefrontal cortex. Neuron 83, 1002–1018. doi: 10.1016/j.neuron.2014.08.011, PMID: 25175878 PMC4156912

[ref158] Szklarczyk D. Franceschini A. Wyder S. Forslund K. Heller D. Huerta-Cepas J.. (2015). STRING v10: protein-protein interaction networks, integrated over the tree of life. Nucleic Acids Res. 43, D447–D452. doi: 10.1093/nar/gku1003 25352553 PMC4383874

[ref159] Tajima A Hans FJ Livingstone D Wei L Finnegan W DeMaro J. Smaller local brain volumes and cerebral atrophy in spontaneously hypertensive rats. Hypertens Dallas Tex (1979). 1993;21:105–111, doi: 10.1161/01.HYP.21.1.105 8418018

[ref160] Taş S. Sarandöl E. Dirican M. (2014). Vitamin B6 supplementation improves oxidative stress and enhances serum paraoxonase/arylesterase activities in streptozotocin-induced diabetic rats. ScientificWorldJournal 2014:351598, 1–7. doi: 10.1155/2014/351598 25431786 PMC4241311

[ref161] Tkachenko H. Kurhaluk N. Grudniewska J. (2014). Oxidative stress biomarkers in different tissues of rainbow trout (*Oncorhynchus mykiss*) exposed to disinfectant-CIP formulated with peracetic acid and hydrogen peroxide. Fish Aquat Life. 22, 207–219. doi: 10.2478/aopf-2014-0021

[ref162] Tsai M. L. Kozłowska A. Li Y. S. Shen W. L. Huang A. C. W. (2017). Social factors affect motor and anxiety behaviors in the animal model of attention-deficit hyperactivity disorders: a housing-style factor. Psychiatry Res. 254, 290–300. doi: 10.1016/j.psychres.2017.05.008, PMID: 28501734

[ref164] van der Meer D. Hoekstra P. J. Bralten J. van Donkelaar M. Heslenfeld D. J. Oosterlaan J.. (2016). Interplay between stress response genes associated with attention-deficit hyperactivity disorder and brain volume. Genes Brain Behav. 15, 627–636. doi: 10.1111/gbb.12307, PMID: 27391809

[ref165] Vašková J. Kočan L. Vaško L. Perjési P. (2023). Glutathione-related enzymes and proteins: a review. Mol. Basel Switz. 28:1447. doi: 10.3390/molecules28031447 PMC9919958 36771108

[ref166] Venkatesan K. Rual J. F. Vazquez A. Stelzl U. Lemmens I. Hirozane-Kishikawa T.. (2009). An empirical framework for binary interactome mapping. Nat. Methods 6, 83–90. doi: 10.1038/nmeth.1280, PMID: 19060904 PMC2872561

[ref167] Verlaet A. A. J. Breynaert A. Ceulemans B. De Bruyne T. Fransen E. Pieters L.. (2019). Oxidative stress and immune aberrancies in attention-deficit/hyperactivity disorder (ADHD): a case-control comparison. Eur. Child Adolesc. Psychiatry 28, 719–729. doi: 10.1007/s00787-018-1239-4, PMID: 30350094

[ref168] Verma P. Singh A. Nthenge-Ngumbau D. N. Rajamma U. Sinha S. Mukhopadhyay K.. (2016). Attention deficit-hyperactivity disorder suffers from mitochondrial dysfunction. BBA Clin. 6, 153–158. doi: 10.1016/j.bbacli.2016.10.003, PMID: 27896136 PMC5121149

[ref169] Wang M. Hu Z. Liu L. Li H. Qian Q. Niu H. (2020). Disrupted functional brain connectivity networks in children with attention-deficit/hyperactivity disorder: evidence from resting-state functional near-infrared spectroscopy. Neurophotonics 7:015012. doi: 10.1117/1.NPh.7.1.015012, PMID: 32206679 PMC7064804

[ref171] Weitner T. Inić S. Jablan J. Gabričević M. Domijan A. M. (2016). Spectrophotometric determination of malondialdehyde in urine suitable for epidemiological studies. Croat. Chem. Acta 89, 133–139. doi: 10.5562/cca2902

[ref172] Welsh K. J. Kirkman M. S. Sacks D. B. (2016). Role of glycated proteins in the diagnosis and Management of Diabetes: research gaps and future directions. Diabetes Care 39, 1299–1306. doi: 10.2337/dc15-2727, PMID: 27457632 PMC4955935

[ref173] Willing J. Juraska J. M. (2015). The timing of neuronal loss across adolescence in the medial prefrontal cortex of male and female rats. Neuroscience 301, 268–275. doi: 10.1016/j.neuroscience.2015.05.073, PMID: 26047728 PMC4504753

[ref174] Wong R. S. Y. (2022). Psychopathology of attention deficit/hyperactivity disorder: from an inflammatory perspective. Egypt J. Neurol. Psychiatry Neurosurg. 58:123. doi: 10.1186/s41983-022-00561-y

[ref175] Wu L. H. Cheng W. Yu M. He B. M. Sun H. Chen Q.. (2017). Nr3C1-Bhlhb2 Axis dysregulation is involved in the development of attention deficit hyperactivity. Mol. Neurobiol. 54, 1196–1212. doi: 10.1007/s12035-015-9679-z, PMID: 26820676 PMC5310568

[ref176] Xiang P. Correlation between interleukin-1β,interleukin-6,tumor necrosis factor-α and attention deficit hyperactivity disorder. (2012) Available at: https://www.semanticscholar.org/paper/Correlation-between-necrosis-factor-%CE%B1-and-attention-Xiang/5666cacf4072d8c72c3af2db8989d4bc5cb15bf3

[ref177] Xue X. Liu B. Hu J. Bian X. Lou S. (2022). The potential mechanisms of lactate in mediating exercise-enhanced cognitive function: a dual role as an energy supply substrate and a signaling molecule. Nutr. Metab. 19:52. doi: 10.1186/s12986-022-00687-z, PMID: 35907984 PMC9338682

[ref178] Yang J. Wang M. Wang S. Li G. Gao Y. (1993). Study on ferroptosis pathway that operates in hypertensive brain damage. Clin. Exp. Hypertens. 42, 748–752. doi: 10.1080/10641963.2020.1783545 32564622

[ref179] Yang Y. Zhang Q. Ren J. Zhu Q. Wang L. Zhang Y.. (2021). Evolution of brain morphology in spontaneously hypertensive and Wistar-Kyoto rats from early adulthood to aging: a longitudinal magnetic resonance imaging study. Front. Aging Neurosci. 13:757808. doi: 10.3389/fnagi.2021.757808, PMID: 34916922 PMC8670306

[ref180] Yde Ohki C. M. Grossmann L. Alber E. Dwivedi T. Berger G. Werling A. M.. (2020). The stress-Wnt-signaling axis: a hypothesis for attention-deficit hyperactivity disorder and therapy approaches. Transl. Psychiatry 10:315. doi: 10.1038/s41398-020-00999-9, PMID: 32948744 PMC7501308

[ref181] Zalcman S. Green-Johnson J. M. Murray L. Nance D. M. Dyck D. Anisman H.. (1994). Cytokine-specific central monoamine alterations induced by interleukin-1, −2 and −6. Brain Res. 643, 40–49. doi: 10.1016/0006-8993(94)90006-X, PMID: 7518332

[ref182] Zametkin A. J. Nordahl T. E. Gross M. King A. C. Semple W. E. Rumsey J.. (1990). Cerebral glucose metabolism in adults with hyperactivity of childhood onset. N. Engl. J. Med. 323, 1361–1366. doi: 10.1056/NEJM199011153232001, PMID: 2233902

[ref183] Zhang W. Yan Z. Gao J. H. Sun L. Huang X. Y. Liu Z.. (2014). Role and mechanism of microglial activation in iron-induced selective and progressive dopaminergic neurodegeneration. Mol. Neurobiol. 49, 1153–1165. doi: 10.1007/s12035-013-8586-4, PMID: 24277523 PMC4878835

[ref184] Zhou R. Y. Wang J. J. You Y. Sun J. C. Song Y. C. Yuan H. X.. (2017). Effect of baicalin on ATPase and LDH and its regulatory effect on the AC/cAMP/PKA signaling pathway in rats with attention deficit hyperactivity disorder. Chin. J. Contemp. Pediatr. 19, 576–582. doi: 10.7499/j.issn.1008-8830.2017.05.020 PMC7389122 28506353

[ref185] Zia A. Pourbagher-Shahri A. M. Farkhondeh T. Samarghandian S. (2021). Molecular and cellular pathways contributing to brain aging. Behav. Brain Funct. 17:6. doi: 10.1186/s12993-021-00179-9, PMID: 34118939 PMC8199306

