# Supplementary material for: Age-related changes in the architecture and biochemical markers levels in motor-related cortical areas of SHR rats—an ADHD animal model
Source: Front Mol Neurosci. 2024 Aug 23;17:1414457. doi: 10.3389/fnmol.2024.1414457 (PMC11378348; doi:10.3389/fnmol.2024.1414457)
Supplement: Supplementary file 1 [file Data_Sheet_1.pdf]

Supplementary material 1

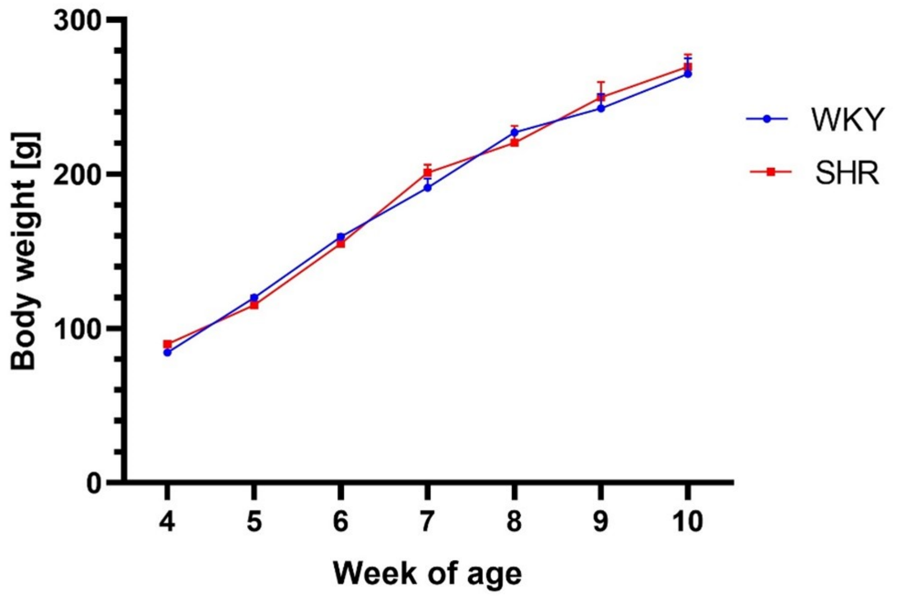

Figure S1. Rats' body weights [g] during the experimental period (4-10 weeks). The two-way ANOVA analysis followed by the Bonferroni post hoc test did not show significant differences between strains (n = 5 or 6 rats per group; mean value ± SD).

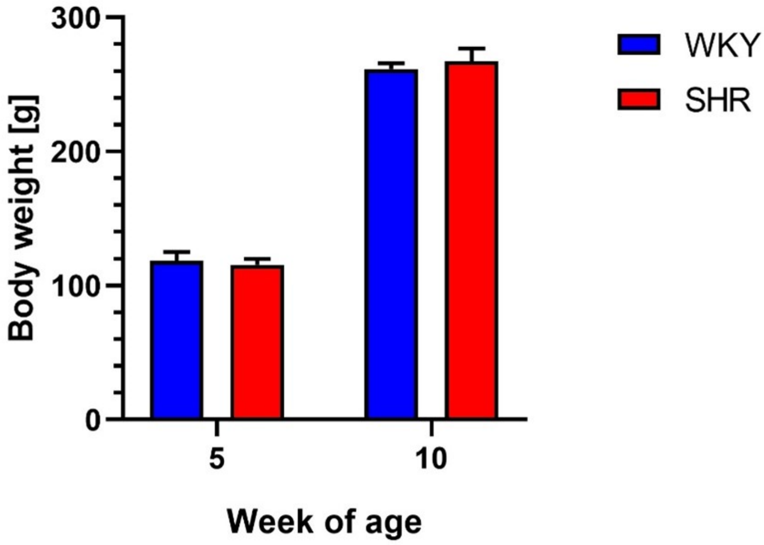

Figure S2. Rats' body weights [g] at 5 and 10 weeks of age. The two-way ANOVA analysis followed by the Bonferroni post hoc test did not show significant differences between strains (n = 6 rats per group; mean value ± SD).
