# Supplementary material for: Age-related changes in the architecture and biochemical markers levels in motor-related cortical areas of SHR rats—an ADHD animal model
Source: Front Mol Neurosci. 2024 Aug 23;17:1414457. doi: 10.3389/fnmol.2024.1414457 (PMC11378348; doi:10.3389/fnmol.2024.1414457)
Supplement: Supplementary file 2 [file Data_Sheet_2.pdf]

Supplementary material 2

Initially, the cytokine panel for this study was meticulously selected through a comprehensive review of the existing literature. The relationship between interleukin-1 alpha (IL-1α), IL-1β, IL-6, mammalian target of rapamycin (mTOR), protein kinase B (AKT-1), and the glucocorticoid receptor β (GCsRβ) is complex and multifaceted, as they are involved in various cellular processes, including inflammation and immune regulation [1]. In brief, IL-1α and IL-1β are pro-inflammatory cytokines known for their capacity to activate immune responses and contribute to inflammation. The downstream effects of IL-1α and IL-1β can lead to the expression of other cytokines, including IL-6 [2] which is produced by diverse cell types, including immune cells and glial cells within the brain and plays a significant role in the acute-phase response and possesses the capability to stimulate the immune system [3]. Furthermore, in response to pro-inflammatory cytokines (IL-1, IL-6, and TNF-α) the intracellular PI3K/AKT/mTOR signaling pathway is activated. Within this pathway, AKT-1 and mTOR serve as two pivotal signaling proteins, regulating a wide spectrum of crucial functions related to neuronal activity, growth, plasticity, and survival [4]. Furthermore, it is important to emphasise that the GCsRβ plays a significant anti-inflammatory effects role in the regulation of neuroinflammation in the central nervous system [5]. The GCsRβ activation in response to glucocorticoid hormones exerts anti-inflammatory effects by reducing the production of pro-inflammatory mediators, it can prevent neuronal damage and death associated with neuroinflammatory conditions [6]. GCsRβ activation is also involved in regulating neurogenesis and synaptic plasticity. These processes are vital for the adaptation and recovery of the brain in the face of neuroinflammatory insults [7]. The available literature highlights dysregulation of the GCsRβ and the Hypothalamic–pituitary–adrenal axis has been associated with a range of neurological and neuropsychiatric disorders, including depression, anxiety, neurodegenerative diseases and also attention deficit hyperactivity disorder. In these conditions, neuroinflammation often plays a contributory role [8–10].

Neuroinflammation and oxidative stress are closely intertwined processes in the CNS, and their interaction carries implications for brain condition and the development of various neurological disorders. As part of the immune defense mechanism, neuroinflammation can initiate the production of reactive oxygen species (ROS). However, excessive ROS production can lead to oxidative stress [11]. Oxidative stress is characterized by an imbalance between ROS generation and the body's capacity to counteract them with antioxidants. This imbalance can further intensify neuroinflammation [12]. ROS have the ability to activate pro-inflammatory signaling pathways and induce the release of additional pro-inflammatory cytokines and chemokines, thus establishing a self-perpetuating cycle of inflammation [13].

In the assessment of oxidative stress in tissues, scientists often use the measurement of specific biochemical markers/enzymes i.e., malondialdehyde (MDA), sulfhydryl (-SH) groups, superoxide dismutase (SOD), peroxidase (POD), glutathione reductase (GSR) and glutathione S-transferase (GST). A concise overview of the activity of the listed markers/enzymes is provided in the table below (Tab. S1).

Table S1. The table succinctly outlines the characterization of the oxidative stress markers/enzymes under investigation: MDA, -SH, SOD, POD, GSR, and GST.

MARKER/ENZYME	ACTIVITY
MDA	Product of lipid peroxidation and is used as a marker to assess oxidative stress and cellular damage [14].
-SH	Groups important in redox reactions and maintaining cellular redox balance; serve as electron donors to mitigate the effects of ROS and free radicals [15].

SOD	An enzyme responsible for catalyzing the dismutation of superoxide radicals (O_2--) into molecular oxygen (O_2) and hydrogen peroxide (H_2O_2). Plays a key role in reducing oxidative stress by neutralising superoxide radicals [16].
POD	A family of enzymes that catalyze the reduction of H_2O_2 and other peroxides. They serve a function in cellular protection from oxidative damage through the breakdown of peroxides [17].
GSR	An enzyme responsible for the regeneration of reduced glutathione (GSH) from its oxidized form (GSSG). This enzyme is essential for maintaining the cellular pool of reduced glutathione, which acts as a powerful antioxidant and helps protect cells from oxidative damage [18].
GST	A family of enzymes that catalyze the conjugation of glutathione (GSH) to various electrophilic compounds, making them more water-soluble and easier to eliminate from the body. GSTs are involved in detoxification processes and play a role in protecting cells from toxic compounds [19].

Current literature underscores a crucial interplay among neuroinflammation, oxidative stress, and metabolic dysfunction in neurons [20]. Oxidative stress, stemming from an imbalance between ROS and antioxidants, can detrimentally impact mitochondria, resulting in decreased ATP production and disrupted energy metabolism [21]. Concurrently, neuroinflammation, characterized by the release of pro-inflammatory cytokines, can perturb signaling pathways, neurotransmitter metabolism, and synaptic function, thereby influencing neuronal metabolic equilibrium [12]. Furthermore, both processes have the capacity to modify enzyme activity, interfering with essential metabolic reactions and perturbing the fine balance between anabolic and catabolic pathways. Oxidative damage to cellular components, including proteins and lipids, further compounds the impairment of metabolic processes [20,22]. Stress-response pathways, activated in response to oxidative stress, may also engender metabolic adaptations aimed at promoting cellular survival. Conversely, altered neuronal metabolism can significantly impact neuroinflammation and oxidative stress in the brain [23]. Disturbed glucose metabolism, such as impaired glucose uptake or utilization, can give rise to energy deficits within neurons, leading to the release of pro-inflammatory cytokines and ROS [24]. Consequently, these metabolic disturbances play a pivotal role in the development of neurodegenerative diseases and various neurological disorders [25]. In our present study, we sought to examine a range of pivotal metabolic markers and enzymes, specifically glucose (G), fructosamine (FrAm), iron (Fe), lactic acid (LA), lactate dehydrogenase (LDH), alanine transaminase (ALT), and aspartate transaminase (AST) have been detailed in the table below (Tab. S2).

Table S2. The table provides a concise characterization of the metabolic markers/enzymes under scrutiny, namely G, FrAm, Fe, LA, LDH, ALT, and AST.

MARKER/ENZYME	FUNCTION
G	The primary energy source in neurons, supporting neurotransmitter production, antioxidant defense, and essential processes for signal transmission and structural integrity [26].
FrAm	It is a product of protein glycation (covalent attachment of sugar to protein). FrAm is formed during G overload and is mainly used as a marker to assess long-term G control [27].
Fe	It serves critical roles in generating energy (ATP), oxygen transport, neurotransmitter synthesis, myelin production, antioxidant defense as well as DNA maintenance (synthesis and repair) [28].
LA	An alternative neuronal energy source, supports metabolic cooperation between neurons and astrocytes, and helps maintain cellular redox balance [29].

LDH	The enzyme responsible for interconverting pyruvate and lactate, allowing neurons to adapt to changing metabolic conditions and energy demands. A non-specific marker of cell injury [30,31].
ALT	Its primary role is to catalyze the conversion of alanine to pyruvate, an important step in G metabolism. Cell and tissue injury marker [32].
AST	It plays a crucial role in amino acid metabolism by catalyzing the conversion of aspartate and alpha-ketoglutarate to oxaloacetate and glutamate. Cell and tissue injury marker [32].

Table S3. List of ELISA kits used for the determination of studied immune markers concentrations in rat PFC.

**Antigen**	**ELISA test**	**Catalogue number**	**Manufacturer, country**
IL-1α	ELISA Kit for Rat IL-1 alfa	E0071r	EIAab, China
IL-1β	ELISA Kit for Rat IL-1 beta	E0563r	EIAab, China
IL-6	ELISA Kit for Rat IL-6	E0079r	EIAab, China
mTOR	ELISA kit for Rat Serine/threonine-protein mTOR,	ER1520	Wuhan Fine Biotech Co, Ltd, China
GCsRβ	ELISA kit for rat Glucocorticoid receptor β	E1608r	EIAab, China
AKT-1	ELISA Kit FOR rat RAC-alpha serine/threonine-protein kinase	E0382r	EIAab, China

Table S4. List of specific sets used to determine studied metabolic markers concentrations in rat PFC.

**Substance**	**Reagent set**	**Catalogue number**	**Manufacturer, country**
G	Glucose oxidase reagent set	G7521	Pointe Scientific, Inc., Poland
FrAm	Fructosamine reagent set	F7546	Pointe Scientific, Inc., Poland
Fe	Total iron reagent set	17505	Pointe Scientific, Inc., Poland
LA	Lactate acid reagent set	L7596	Pointe Scientific, Inc., Poland

Table S5. List of specific sets used to determine studied oxidative stress markers concentrations in rat PFC.

Enzyme	Reagent set	Catalogue number	Manufacturer, country
ALT	Alanine transaminase (SGPT) reagent set	A7526	Pointe Scientific, Inc., Poland
AST	Aspartate aminotransferase (SGOT) reagent set	A7561	Pointe Scientific, Inc., Poland
LDH	Lactate dehydrogenase reagent set	L7572	Pointe Scientific, Inc., Poland

References:

1. Beck, I. M. E. *et al.* Crosstalk in Inflammation: The Interplay of Glucocorticoid Receptor-Based Mechanisms and Kinases and Phosphatases. *Endocr. Rev.* **30**, 830–882 (2009).
2. Lopez-Castejon, G. & Brough, D. Understanding the mechanism of IL-1β secretion. *Cytokine Growth Factor Rev.* **22**, 189–195 (2011).
3. Erta, M., Quintana, A. & Hidalgo, J. Interleukin-6, a Major Cytokine in the Central Nervous System. *Int. J. Biol. Sci.* **8**, 1254–1266 (2012).
4. Sánchez-Alegría, K., Flores-León, M., Avila-Muñoz, E., Rodríguez-Corona, N. & Arias, C. PI3K Signaling in Neurons: A Central Node for the Control of Multiple Functions. *Int. J. Mol. Sci.* **19**, 3725 (2018).
5. Pace, T. W. W. & Miller, A. H. Cytokines and glucocorticoid receptor signaling. Relevance to major depression. *Ann. N. Y. Acad. Sci.* **1179**, 86–105 (2009).
6. Koning, A.-S. C. A. M., Buurstede, J. C., van Weert, L. T. C. M. & Meijer, O. C. Glucocorticoid and Mineralocorticoid Receptors in the Brain: A Transcriptional Perspective. *J. Endocr. Soc.* **3**, 1917–1930 (2019).
7. Myers, B., McKlveen, J. M. & Herman, J. P. Glucocorticoid actions on synapses, circuits, and behavior: Implications for the energetics of stress. *Front. Neuroendocrinol.* **35**, 180–196 (2014).
8. Du, X. & Pang, T. Y. Is Dysregulation of the HPA-Axis a Core Pathophysiology Mediating Co-Morbid Depression in Neurodegenerative Diseases? *Front. Psychiatry* **6**, 32 (2015).

9. Misiak, B. *et al.* The HPA axis dysregulation in severe mental illness: Can we shift the blame to gut microbiota? *Prog. Neuropsychopharmacol. Biol. Psychiatry* **102**, 109951 (2020).
10. Pinto, R. *et al.* The aetiological association between the dynamics of cortisol productivity and ADHD. *J. Neural Transm.* **123**, 991–1000 (2016).
11. Fabisiak, T. & Patel, M. Crosstalk between neuroinflammation and oxidative stress in epilepsy. *Front. Cell Dev. Biol.* **10**, 976953 (2022).
12. Corona, J. C. Role of Oxidative Stress and Neuroinflammation in Attention-Deficit/Hyperactivity Disorder. *Antioxidants* **9**, 1039 (2020).
13. Mahmoud, A. M., Wilkinson, F. L., Sandhu, M. A. & Lightfoot, A. P. The Interplay of Oxidative Stress and Inflammation: Mechanistic Insights and Therapeutic Potential of Antioxidants. *Oxid. Med. Cell. Longev.* **2021**, e9851914 (2021).
14. Bulut, M. *et al.* Malondialdehyde levels in adult attention-deficit hyperactivity disorder. *J. Psychiatry Neurosci. JPN* **32**, 435–438 (2007).
15. Baba, S. P. & Bhatnagar, A. ROLE OF THIOLS IN OXIDATIVE STRESS. *Curr. Opin. Toxicol.* **7**, 133–139 (2018).
16. Boriskin, P., Gulenko, O., Deviatkin, A., Pavlova, O. & Toropovskiy, A. Correlation of superoxide dismutase activity distribution in serum and tissues of small experimental animals. *IOP Conf. Ser. Earth Environ. Sci.* **403**, 012112 (2019).
17. Nandi, A., Yan, L.-J., Jana, C. K. & Das, N. Role of Catalase in Oxidative Stress- and Age-Associated Degenerative Diseases. *Oxid. Med. Cell. Longev.* **2019**, 1–19 (2019).
18. Pizzorno, J. Glutathione! *Integr. Med. Clin. J.* **13**, 8–12 (2014).
19. Habig, W. H., Pabst, M. J. & Jakoby, W. B. Glutathione S-transferases. The first enzymatic step in mercapturic acid formation. *J. Biol. Chem.* **249**, 7130–7139 (1974).
20. Picca, A. *et al.* Mitochondrial Dysfunction, Oxidative Stress, and Neuroinflammation: Intertwined Roads to Neurodegeneration. *Antioxidants* **9**, 647 (2020).

21. Guo, C., Sun, L., Chen, X. & Zhang, D. Oxidative stress, mitochondrial damage and neurodegenerative diseases. *Neural Regen. Res.* **8**, 2003–2014 (2013).

22. Balogh, E. *et al.* Oxidative stress impairs energy metabolism in primary cells and synovial tissue of patients with rheumatoid arthritis. *Arthritis Res. Ther.* **20**, 95 (2018).

23. Song, Y. *et al.* An Shen Ding Zhi Ling Alleviates Symptoms of Attention Deficit Hyperactivity Disorder via Anti-Inflammatory Effects in Spontaneous Hypertensive Rats. *Front. Pharmacol.* **11**, (2021).

24. González, P., Lozano, P., Ros, G. & Solano, F. Hyperglycemia and Oxidative Stress: An Integral, Updated and Critical Overview of Their Metabolic Interconnections. *Int. J. Mol. Sci.* **24**, 9352 (2023).

25. Duarte, J. M. N., Schuck, P. F., Wenk, G. L. & Ferreira, G. C. Metabolic Disturbances in Diseases with Neurological Involvement. *Aging Dis.* **5**, 238–255 (2013).

26. Zhang, S., Lachance, B. B., Mattson, M. P. & Jia, X. Glucose metabolic crosstalk and regulation in brain function and diseases. *Prog. Neurobiol.* **204**, 102089 (2021).

27. Armbruster, D. A. Fructosamine: structure, analysis, and clinical usefulness. *Clin. Chem.* **33**, 2153–2163 (1987).

28. Chen, P. *et al.* Iron and manganese-related CNS toxicity: mechanisms, diagnosis and treatment. *Expert Rev. Neurother.* **19**, 243–260 (2019).

29. Xue, X., Liu, B., Hu, J., Bian, X. & Lou, S. The potential mechanisms of lactate in mediating exercise-enhanced cognitive function: a dual role as an energy supply substrate and a signaling molecule. *Nutr. Metab.* **19**, 52 (2022).

30. Magistretti, P. J. & Allaman, I. Lactate in the brain: from metabolic end-product to signalling molecule. *Nat. Rev. Neurosci.* **19**, 235–249 (2018).

31. Long, D. M. *et al.* Lactate dehydrogenase expression modulates longevity and neurodegeneration in Drosophila melanogaster. *Aging* **12**, 10041–10058 (2020).

32. McGill, M. R. The past and present of serum aminotransferases and the future of liver injury biomarkers. *EXCLI J.* **15**, 817–828 (2016).
