# Supplementary material for: Age-related changes in the architecture and biochemical markers levels in motor-related cortical areas of SHR rats—an ADHD animal model
Source: Front Mol Neurosci. 2024 Aug 23;17:1414457. doi: 10.3389/fnmol.2024.1414457 (PMC11378348; doi:10.3389/fnmol.2024.1414457)
Supplement: Supplementary file 3 [file Data_Sheet_3.pdf]

# Supplementary material 3

**Table S1.** Results of the two-way ANOVA for volume of M1.

| VOLUME OF M1 | | |
|---|---|---|
| **ANOVA table** | **F (DFn. DFd)** | **P value** |
| Interaction | $F_{(18.\ 132)} = 3.168$ | p<0.0001 |
| Row Factor | $F_{(6.\ 132)} = 52.90$ | p<0.0001 |
| Column Factor | $F_{(3.\ 132)} = 160.6$ | p<0.0001 |

**Table S2.** Results of the two-way ANOVA for volume of M2.

| VOLUME OF M2 | | |
|---|---|---|
| **ANOVA table** | **F (DFn. DFd)** | **P value** |
| Interaction | $F_{(18.\ 132)} = 1.923$ | p=0.0189 |
| Row Factor | $F_{(6.\ 132)} = 43.29$ | p<0.0001 |
| Column Factor | $F_{(3.\ 132)} = 123.2$ | p<0.0001 |

**Table S3.** Results of the two-way ANOVA for number of neurons of M1.

| Number of neurons: M1 layer I | | |
|---|---|---|
| **ANOVA table** | **F (DFn. DFd)** | **P value** |
| Interaction | $F_{(18.\ 132)} = 3.030$ | P=0.0001 |
| Row Factor | $F_{(6.\ 132)} = 26.99$ | P<0.0001 |
| Column Factor | $F_{(3.\ 132)} = 1.141$ | P=0.3350 |
| **Number of neurons: M1 layer II** | | |
| **ANOVA table** | **F (DFn. DFd)** | **P value** |
| Interaction | $F_{(18.\ 132)} = 24.81$ | p<0.0001 |
| Row Factor | $F_{(6.\ 132)} = 52.79$ | p<0.0001 |
| Column Factor | $F_{(3.\ 132)} = 21.95$ | p<0.0001 |
| **Number of neurons: M1 layer III** | | |
| **ANOVA table** | **F (DFn. DFd)** | **P value** |
| Interaction | $F_{(18.\ 132)} = 42.26$ | p<0.0001 |
| Row Factor | $F_{(6.\ 132)} = 122.8$ | p<0.0001 |
| Column Factor | $F_{(3.\ 132)} = 19.26$ | p<0.0001 |
| **Number of neurons: M1 layer IV** | | |
| **ANOVA table** | **F (DFn. DFd)** | **P value** |
| Interaction | $F_{(18.\ 132)} = 14.08$ | p<0.0001 |
| Row Factor | $F_{(6.\ 132)} = 71.20$ | p<0.0001 |
| Column Factor | $F_{(3.\ 132)} = 6.001$ | p=0.0007 |
| **Number of neurons: M1 layer V** | | |
| **ANOVA table** | **F (DFn. DFd)** | **P value** |
| Interaction | $F_{(18.\ 132)} = 23.84$ | p<0.0001 |
| Row Factor | $F_{(6.\ 132)} = 59.37$ | p<0.0001 |
| Column Factor | $F_{(3.\ 132)} = 11.35$ | p<0.0001 |
| **Number of neurons: M1 layer VI** | | |
| **ANOVA table** | **F (DFn. DFd)** | **P value** |
| Interaction | $F_{(18.\ 132)} = 8.915$ | p<0.0001 |
| Row Factor | $F_{(6.\ 132)} = 61.12$ | p<0.0001 |

| | | |
|---|---|---|
| Column Factor | $F_{(3. 132)} = 28.44$ | p<0.0001 |

**Table S4.** Results of the two-way ANOVA for number of neurons of M2.

| **Number of neurons: M2 layer I** | | |
|---|---|---|
| **ANOVA table** | **F (DFn. DFd)** | **P value** |
| Interaction | $F_{(18. 132)} = 5.884$ | p<0.0001 |
| Row Factor | $F_{(6. 132)} = 40.48$ | p<0.0001 |
| Column Factor | $F_{(3. 132)} = 5.701$ | p=0.0011 |
| **Number of neurons: M2 layer II** | | |
| **ANOVA table** | **F (DFn. DFd)** | **P value** |
| Interaction | $F_{(18. 132)} = 23.28$ | p<0.0001 |
| Row Factor | $F_{(6. 132)} = 59.23$ | p<0.0001 |
| Column Factor | $F_{(3. 132)} = 10.28$ | p<0.0001 |
| **Number of neurons: M2 layer III** | | |
| **ANOVA table** | **F (DFn. DFd)** | **P value** |
| Interaction | $F_{(18. 132)} = 34.23$ | p<0.0001 |
| Row Factor | $F_{(6. 132)} = 82.84$ | p<0.0001 |
| Column Factor | $F_{(3. 132)} = 26.36$ | p<0.0001 |
| **Number of neurons: M2 layer IV** | | |
| **ANOVA table** | **F (DFn. DFd)** | **P value** |
| Interaction | $F_{(18. 132)} = 17.85$ | P<0.0001 |
| Row Factor | $F_{(6. 132)} = 76.37$ | P<0.0001 |
| Column Factor | $F_{(3. 132)} = 2.939$ | P=0.0356 |
| **Number of neurons: M2 layer V** | | |
| **ANOVA table** | **F (DFn. DFd)** | **P value** |
| Interaction | $F_{(18. 132)} = 16.74$ | p<0.0001 |
| Row Factor | $F_{(6. 132)} = 54.53$ | p<0.0001 |
| Column Factor | $F_{(3. 132)} = 8.497$ | p<0.0001 |
| **Number of neurons: M2 layer VI** | | |
| **ANOVA table** | **F (DFn. DFd)** | **P value** |
| Interaction | $F_{(18. 132)} = 11.27$ | p<0.0001 |
| Row Factor | $F_{(6. 132)} = 103.1$ | p<0.0001 |
| Column Factor | $F_{(3. 132)} = 36.56$ | p<0.0001 |

**Table S5.** Results of the two-way ANOVA for immune markers.

| **IL-α** | | |
|---|---|---|
| **ANOVA table** | **F (DFn. DFd)** | **P value** |
| Interaction | $F_{(1. 20)} = 2.534$ | p=0.1271 |
| Row Factor | $F_{(1. 20)} = 6.441$ | p=0.0196 |
| Column Factor | $F_{(1. 20)} = 6.653$ | p=0.0179 |
| **IL-β** | | |
| **ANOVA table** | **F (DFn. DFd)** | **P value** |
| Interaction | $F_{(1. 20)} = 4.938 \times 10^{-5}$ | p=0.9945 |
| Row Factor | $F_{(1. 20)} = 4.818$ | p=0.0401 |
| Column Factor | $F_{(1. 20)} = 0.6493$ | p=0.4298 |
| **IL-6** | | |
| **ANOVA table** | **F (DFn. DFd)** | **P value** |

| | | |
|---|---|---|
| Interaction | $F_{(1. 20)} = 3.458$ | p=0.0777 |
| Row Factor | $F_{(1. 20)} = 9.500$ | p=0.0059 |
| Column Factor | $F_{(1. 20)} = 8.419$ | p=0.0088 |
| **m-TOR** | | |
| **ANOVA table** | **F (DFn. DFd)** | **P value** |
| Interaction | $F_{(1. 20)} = 1.269$ | p=0.2734 |
| Row Factor | $F_{(1. 20)} = 8.840$ | p=0.0075 |
| Column Factor | $F_{(1. 20)} = 9.084$ | p=0.0069 |
| **GCsRβ** | | |
| **ANOVA table** | **F (DFn. DFd)** | **P value** |
| Interaction | $F_{(1. 20)} = 3.330$ | p=0.0830 |
| Row Factor | $F_{(1. 20)} = 1.599$ | p=0.2206 |
| Column Factor | $F_{(1. 20)} = 10.86$ | p=0.0036 |
| **AKT-1** | | |
| **ANOVA table** | **F (DFn. DFd)** | **P value** |
| Interaction | $F_{(1. 20)} = 6.145$ | p=0.0222 |
| Row Factor | $F_{(1. 20)} = 4.344$ | p=0.0502 |
| Column Factor | $F_{(1. 20)} = 3.973$ | p=0.0600 |

**Table S6.** Results of the two-way ANOVA for oxidative stress markers.

| **MDA** | | |
|---|---|---|
| **ANOVA table** | **F (DFn. DFd)** | **P value** |
| Interaction | $F_{(1. 20)} = 17.21$ | p=0.0005 |
| Row Factor | $F_{(1. 20)} = 3.293$ | p=0.0846 |
| Column Factor | $F_{(1. 20)} = 18.84$ | p=0.0003 |
| **-SH** | | |
| **ANOVA table** | **F (DFn. DFd)** | **P value** |
| Interaction | $F_{(1. 20)} = 7.400$ | p=0.0132 |
| Row Factor | $F_{(1. 20)} = 4.381$ | p=0.0493 |
| Column Factor | $F_{(1. 20)} = 5.784$ | p=0.0260 |
| **SOD** | | |
| **ANOVA table** | **F (DFn. DFd)** | **P value** |
| Interaction | $F_{(1. 20)} = 7.070$ | p=0.0151 |
| Row Factor | $F_{(1. 20)} = 2.909$ | p=0.1036 |
| Column Factor | $F_{(1. 20)} = 6.061$ | p=0.0230 |
| **POD** | | |
| **ANOVA table** | **F (DFn. DFd)** | **P value** |
| Interaction | $F_{(1. 20)} = 2.370$ | p=0.1394 |
| Row Factor | $F_{(1. 20)} = 4.116$ | p=0.0560 |
| Column Factor | $F_{(1. 20)} = 6.807$ | P=0.0168 |
| **GSR** | | |
| **ANOVA table** | **F (DFn. DFd)** | **P value** |
| Interaction | $F_{(1. 20)} = 6.403$ | p=0.0199 |
| Row Factor | $F_{(1. 20)} = 15.66$ | p=0.0008 |
| Column Factor | $F_{(1. 20)} = 6.545$ | p=0.0187 |
| **GST** | | |
| **ANOVA table** | **F (DFn. DFd)** | **P value** |
| Interaction | $F_{(1. 20)} = 6.118$ | P=0.0225 |

| | | |
|---|---|---|
| Row Factor | $F_{(1.\ 20)} = 6.452$ | P=0.0195 |
| Column Factor | $F_{(1.\ 20)} = 4.150$ | P=0.0551 |

**Table S7.** Results of the two-way ANOVA for metabolism markers.

| **G** | | |
|---|---|---|
| **ANOVA table** | **F (DFn. DFd)** | **P value** |
| Interaction | $F_{(1.\ 20)} = 6.324$ | p=0.0206 |
| Row Factor | $F_{(1.\ 20)} = 5.381$ | p=0.0311 |
| Column Factor | $F_{(1.\ 20)} = 2.700$ | p=0.1160 |
| **FrAm** | | |
| **ANOVA table** | **F (DFn. DFd)** | **P value** |
| Interaction | $F_{(1.\ 20)} = 5.156$ | p=0.0344 |
| Row Factor | $F_{(1.\ 20)} = 3.068$ | p=0.0952 |
| Column Factor | $F_{(1.\ 20)} = 3.730$ | p=0.0678 |
| **Fe** | | |
| **ANOVA table** | **F (DFn. DFd)** | **P value** |
| Interaction | $F_{(1.\ 20)} = 5.562$ | p=0.0286 |
| Row Factor | $F_{(1.\ 20)} = 0.3216$ | p=0.5770 |
| Column Factor | $F_{(1.\ 20)} = 3.812$ | p=0.0650 |
| **LA** | | |
| **ANOVA table** | **F (DFn. DFd)** | **P value** |
| Interaction | $F_{(1.\ 20)} = 2.814$ | p=0.1090 |
| Row Factor | $F_{(1.\ 20)} = 0.3244$ | p=0.5753 |
| Column Factor | $F_{(1.\ 20)} = 6.125$ | p=0.0224 |
| **ALT** | | |
| **ANOVA table** | **F (DFn. DFd)** | **P value** |
| Interaction | $F_{(1.\ 20)} = 4.518$ | p=0.0462 |
| Row Factor | $F_{(1.\ 20)} = 4.160$ | p=0.0548 |
| Column Factor | $F_{(1.\ 20)} = 7.218$ | p=0.0142 |
| **AST** | | |
| **ANOVA table** | **F (DFn. DFd)** | **P value** |
| Interaction | $F_{(1.\ 20)} = 10.57$ | p=0.0040 |
| Row Factor | $F_{(1.\ 20)} = 2.350$ | p=0.1409 |
| Column Factor | $F_{(1.\ 20)} = 1.326$ | p=0.2631 |
| **LDH** | | |
| **ANOVA table** | **F (DFn. DFd)** | **P value** |
| Interaction | $F_{(1.\ 20)} = 7.840$ | p=0.0111 |
| Row Factor | $F_{(1.\ 20)} = 3.590$ | p=0.0727 |
| Column Factor | $F_{(1.\ 20)} = 5.128$ | p=0.0348 |
