# Supplementary material for: Age-related changes in the architecture and biochemical markers levels in motor-related cortical areas of SHR rats—an ADHD animal model
Source: Front Mol Neurosci. 2024 Aug 23;17:1414457. doi: 10.3389/fnmol.2024.1414457 (PMC11378348; doi:10.3389/fnmol.2024.1414457)
Supplement: Supplementary file 4 [file Data_Sheet_4.pdf]

Supplementary material 4

Table S1. Network statistics and selected biological process (Gene Ontology) of the interactions

Biological process (Gene Ontology)	Description*	Strenght	p-value
**Social behavior**	Behavior directed towards society or taking place between members of the same species. Occurs predominantly, or only, in individuals that are part of a group.	1.79	1.86×10^{-2}*
**Positive regulation of neuron death**	Any process that activates or increases the frequency, rate or extent of neuron death.	1.65	3.6×10^{-3}**
**Cell aging**	An aging process that has as participant a cell after a cell has stopped dividing.	1.65	2.98×10^{-2}*
**Regulation of behavior**	Any process that modulates the frequency, rate or extent of behavior, the internally coordinated responses (actions or inactions) of whole living organisms (individuals or groups) to internal or external stimuli.	1.55	4.21×10^{-2}*
**Regulation of nervous system process**	Any process that modulates the frequency, rate or extent of a neurophysiological process, an organ system process carried out by any of the organs or tissues of the nervous system.	1.48	7.5×10^{-3}**
**Aging**	A developmental process that is a deterioration and loss of function over time. Aging includes loss of functions such as resistance to disease, homeostasis, and fertility, as well as wear and tear. Aging includes cellular senescence, but is more inclusive.	1.46	5.01×10^{-6}***
**Regulation of neuron death**	Any process that modulates the frequency, rate or extent of neuron death.	1.37	3.9×10^{-4}***
**Positive regulation of neurogenesis**	Any process that activates or increases the frequency, rate or extent of neurogenesis, the generation of cells within the nervous system.	1.08	1.17×10^{-2}*
**Regulation of neuron projection development**	Any process that modulates the rate, frequency or extent of neuron projection development. Neuron projection development is the process whose specific outcome is the progression of a neuron projection over time, from its formation to the mature structure. A neuron projection is any process extending from a neural cell, such as axons or dendrites (collectively called neurites).	1.05	1.35×10^{-2}*

**Behavior**	The internally coordinated responses (actions or inactions) of animals (individuals or groups) to internal or external stimuli, via a mechanism that involves nervous system activity.	0.97	2.33×10^{-2}*
**Regulation of response to stress**	Any process that modulates the frequency, rate or extent of a response to stress. Response to stress is a change in state or activity of a cell or an organism (in terms of movement, secretion, enzyme production, gene expression, etc.) as a result of a disturbance in organismal or cellular homeostasis, usually, but not necessarily, exogenous (e.g. temperature, humidity, ionizing radiation).	0.91	3.6×10^{-3}**
**Generation of neurons**	The process in which nerve cells are generated. This includes the production of neuroblasts and their differentiation into neurons.	0.79	9.1×10^{-3}**
**Response to stress**	Any process that results in a change in state or activity of a cell or an organism (in terms of movement, secretion, enzyme production, gene expression, etc.) as a result of a disturbance in organismal or cellular homeostasis, usually, but not necessarily, exogenous (e.g. temperature, humidity, ionizing radiation).	0.61	7.6×10^{-3}**
**Developmental process**	A biological process whose specific outcome is the progression of an integrated living unit: an anatomical structure (which may be a subcellular structure, cell, tissue, or organ), or organism over time from an initial condition to a later condition.	0.46	8.1×10^{-3}**

*($p \leq 0.05$), **($p \leq 0.01$) and ***($p \leq 0.001$) demonstrates statistically significant. The data have been sorted into decreasing strength.

*Source of all descriptions of the listed biological functions - Gene Ontology (GO) (http://geneontology.org/)
